# Supplementary material for: Gasdermin D in macrophages drives orchitis by regulating inflammation and antigen presentation processes
Source: EMBO Mol Med. 2024 Jan 2;16(2):8. doi: 10.1038/s44321-023-00016-8 (PMC10897472; doi:10.1038/s44321-023-00016-8)
Supplement: Supplementary file 2 — Source Data Fig. 1 [file 44321_2023_16_MOESM2_ESM.zip › Figure1/Figure1C Blot/WB原始图.pptx]

## Slide 1
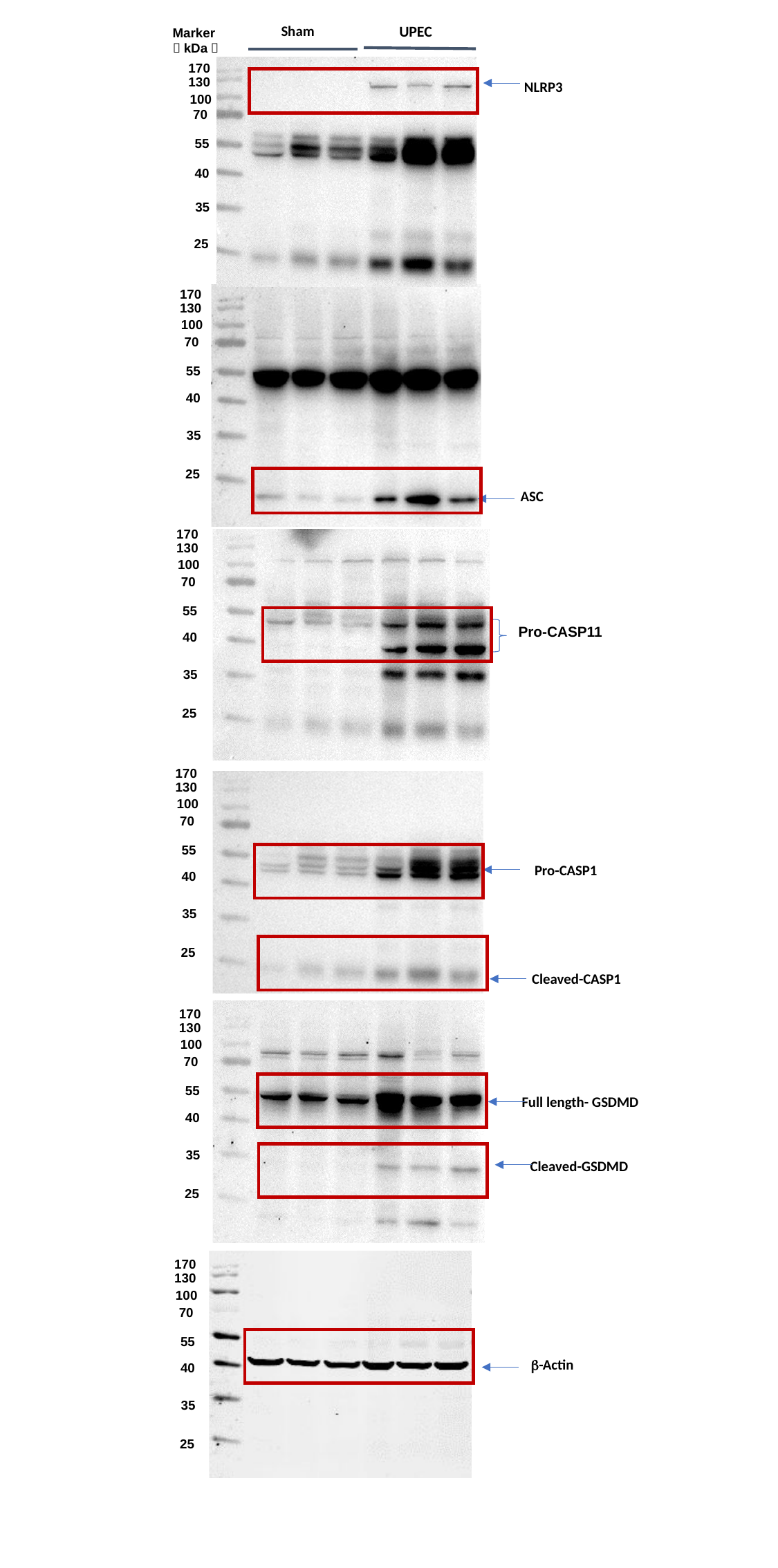

UPEC
Sham
Marker
（kDa）
170
130
100
70
55
40
35
25
NLRP3
170
130
100
70
55
40
35
25
ASC
170
130
100
70
55
40
35
25
Pro-CASP11
170
130
100
70
55
40
35
25
Pro-CASP1
Cleaved-CASP1
170
130
100
70
55
40
35
25
Full length- GSDMD
Cleaved-GSDMD
170
130
100
70
55
40
35
25
b-Actin
