# Supplementary figures and images for: Gasdermin D in macrophages drives orchitis by regulating inflammation and antigen presentation processes
Source: EMBO Mol Med. 2024 Jan 2;16(2):8. doi: 10.1038/s44321-023-00016-8 (PMC10897472; doi:10.1038/s44321-023-00016-8)

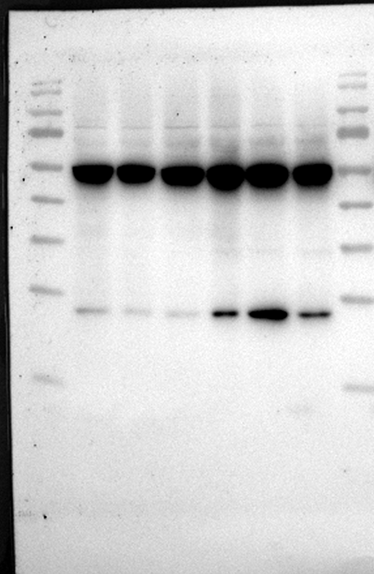

Supplement: Supplementary file 2 — Source Data Fig. 1 [file 44321_2023_16_MOESM2_ESM.zip › Figure1/Figure1C Blot/WETERN ASC.tif]

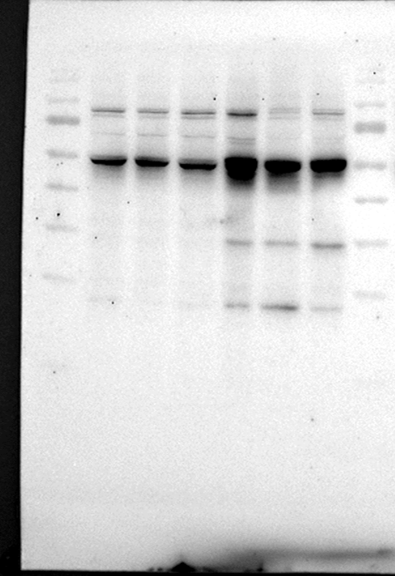

Supplement: Supplementary file 2 — Source Data Fig. 1 [file 44321_2023_16_MOESM2_ESM.zip › Figure1/Figure1C Blot/WETERN GSDMD.tif]

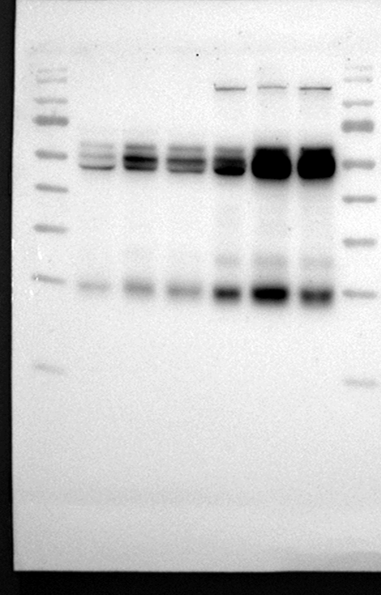

Supplement: Supplementary file 2 — Source Data Fig. 1 [file 44321_2023_16_MOESM2_ESM.zip › Figure1/Figure1C Blot/WETERN NLRP3.tif]

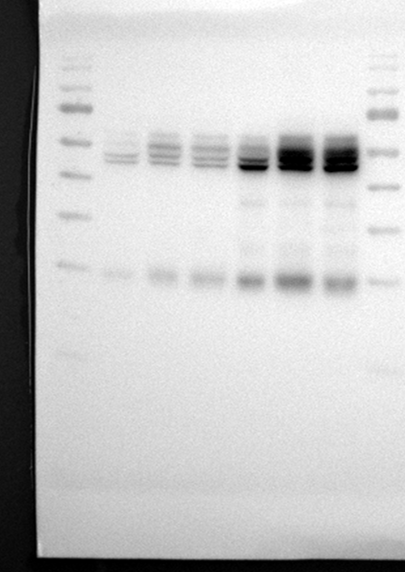

Supplement: Supplementary file 2 — Source Data Fig. 1 [file 44321_2023_16_MOESM2_ESM.zip › Figure1/Figure1C Blot/WETERN PRO-CASPASE1.tif]

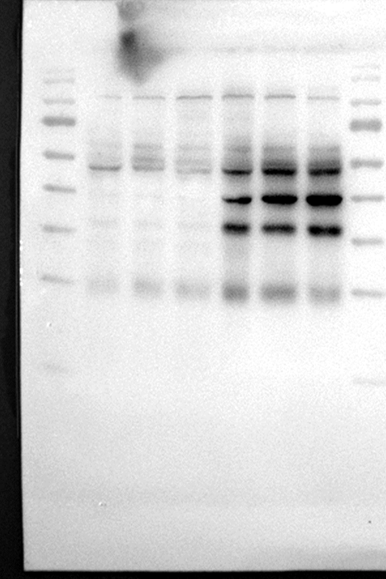

Supplement: Supplementary file 2 — Source Data Fig. 1 [file 44321_2023_16_MOESM2_ESM.zip › Figure1/Figure1C Blot/WETERN PRO-CASPASE11.tif]

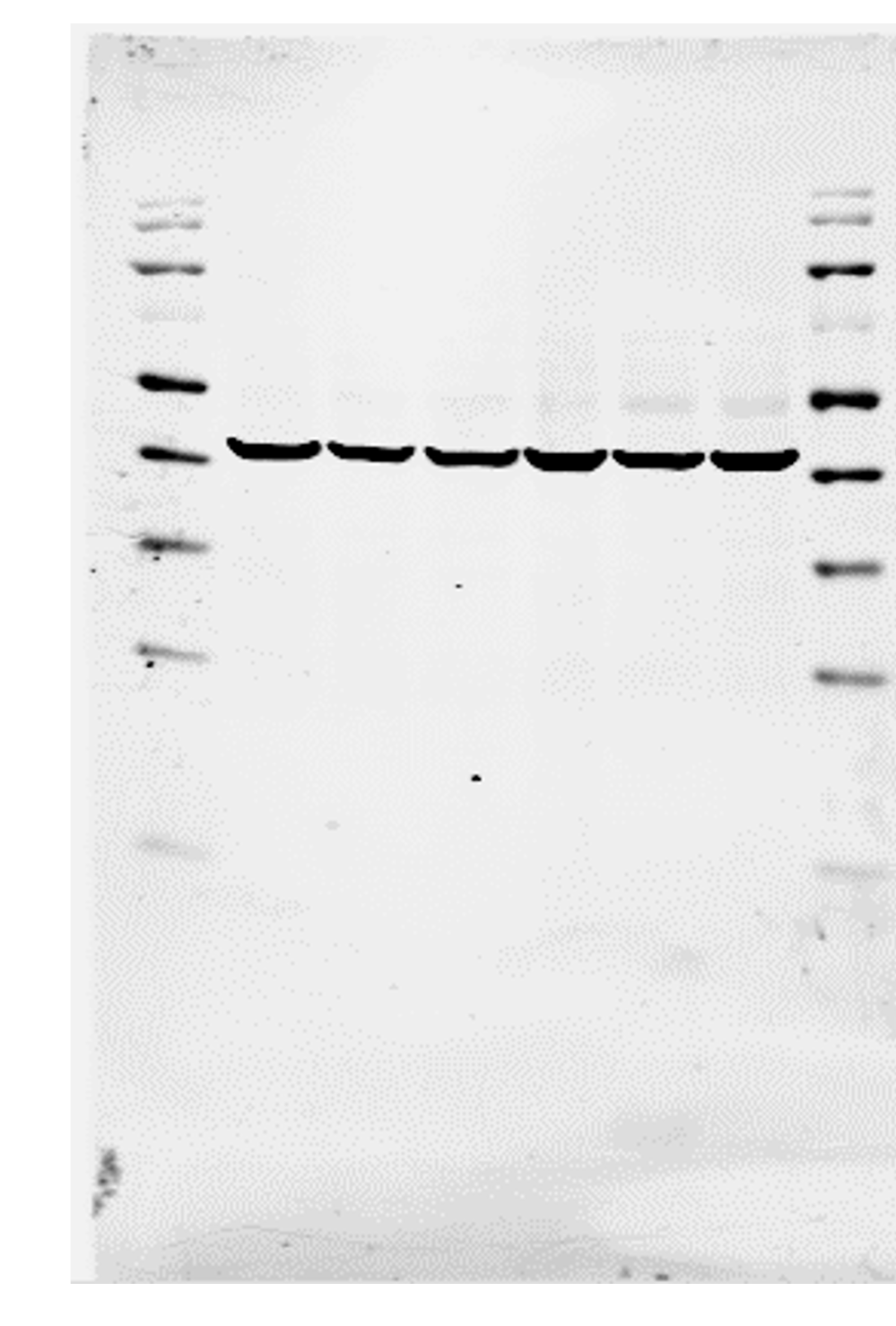

Supplement: Supplementary file 2 — Source Data Fig. 1 [file 44321_2023_16_MOESM2_ESM.zip › Figure1/Figure1C Blot/WETERN PRO-b-Actin.tif]

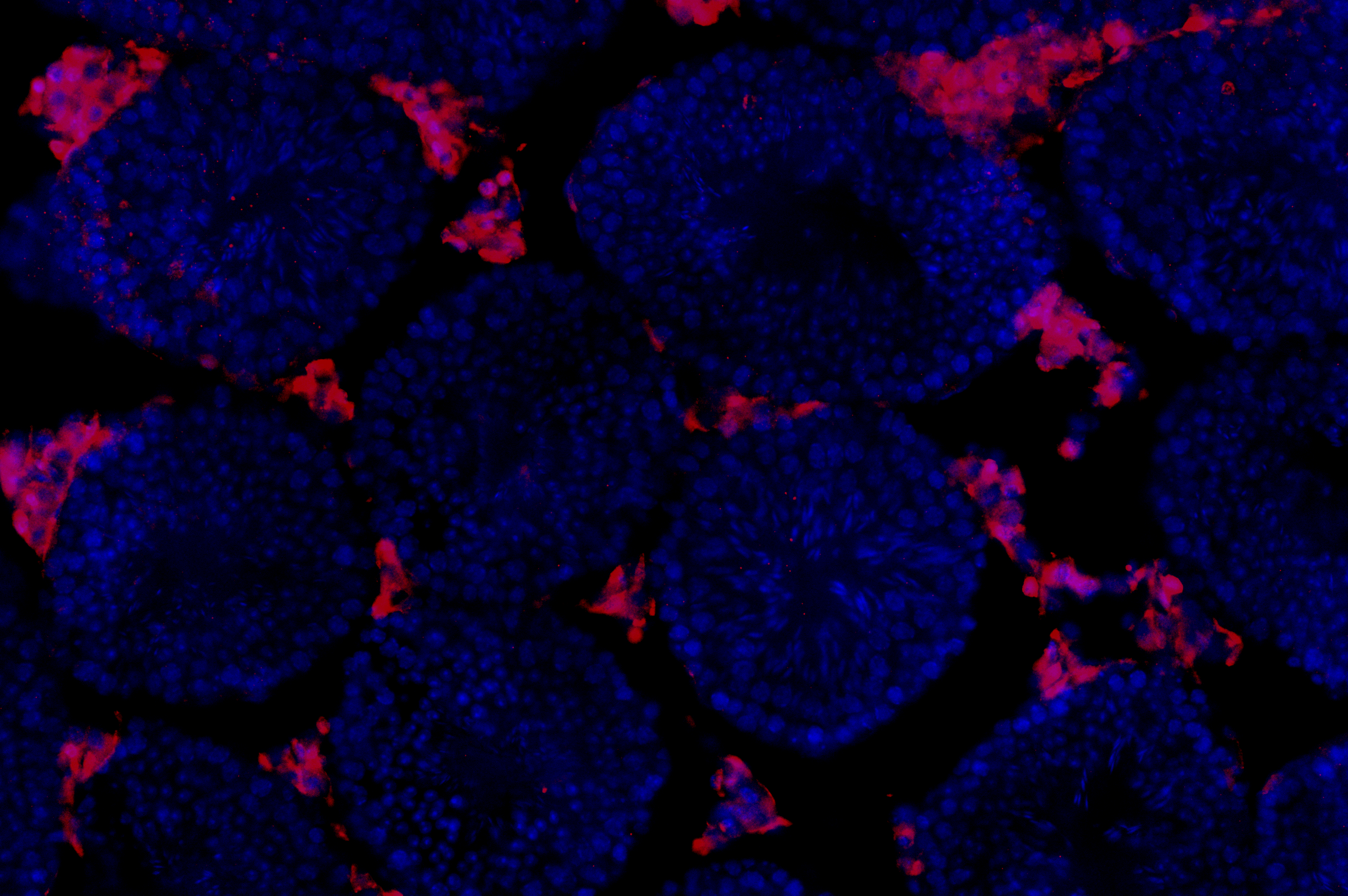

Supplement: Supplementary file 2 — Source Data Fig. 1 [file 44321_2023_16_MOESM2_ESM.zip › Figure1/Figure1D Micr image/GSDMD DAPI UPEC.tif]

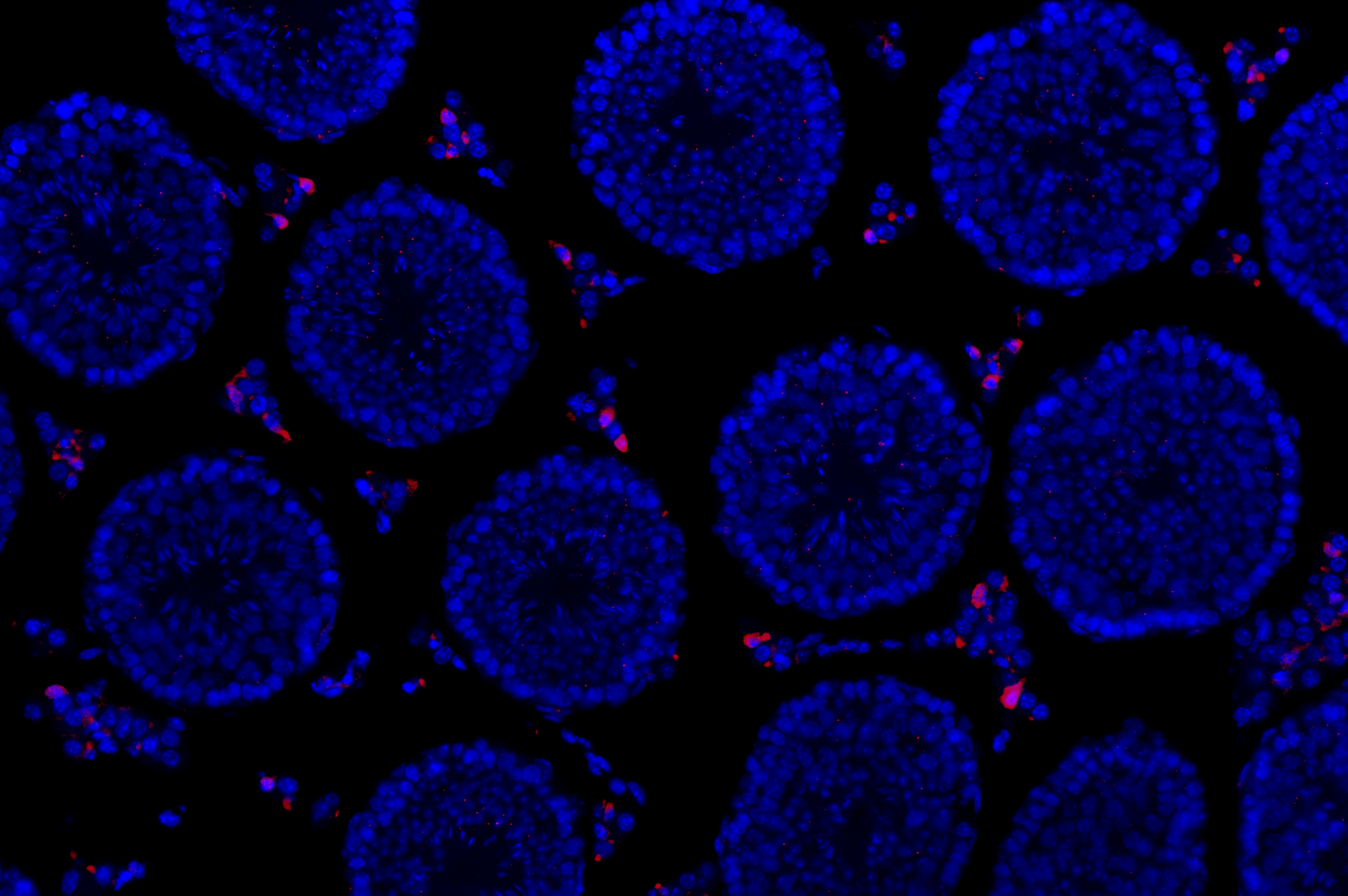

Supplement: Supplementary file 2 — Source Data Fig. 1 [file 44321_2023_16_MOESM2_ESM.zip › Figure1/Figure1D Micr image/GSDMD DAPI sham.tif]

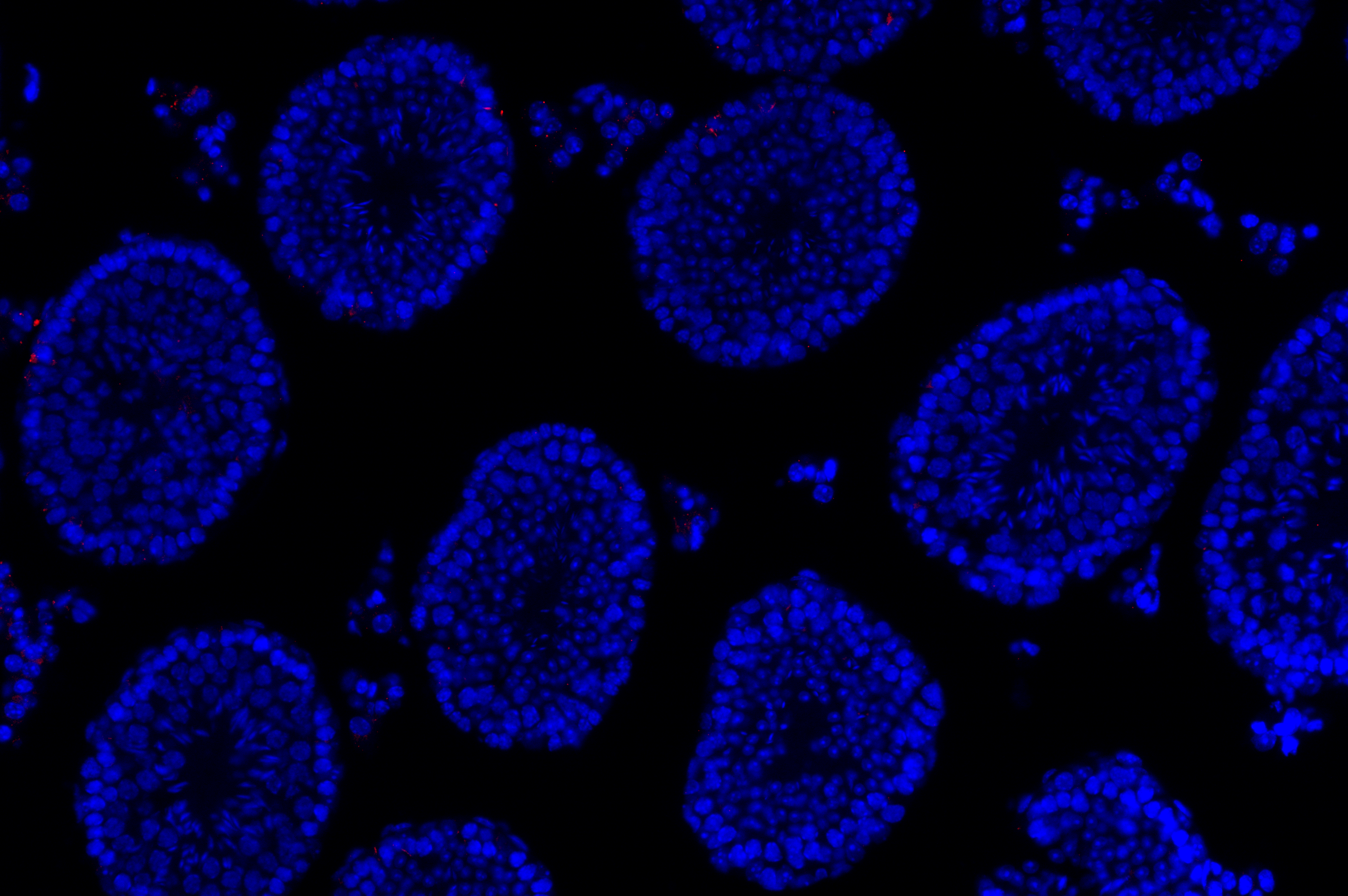

Supplement: Supplementary file 2 — Source Data Fig. 1 [file 44321_2023_16_MOESM2_ESM.zip › Figure1/Figure1D Micr image/NLRP3 DAPI sham.tif]

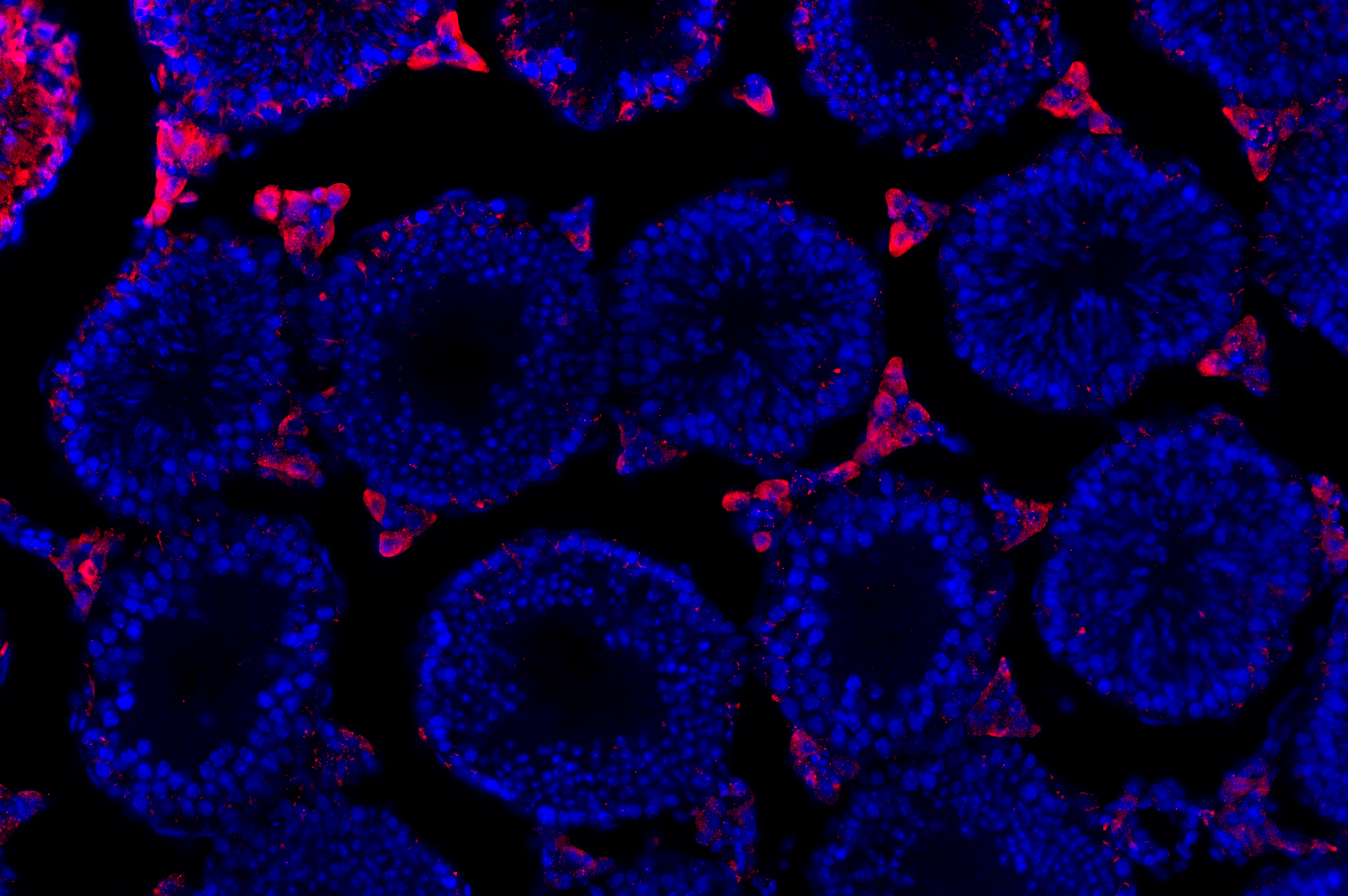

Supplement: Supplementary file 2 — Source Data Fig. 1 [file 44321_2023_16_MOESM2_ESM.zip › Figure1/Figure1D Micr image/NLRP3 DAPI upec.tif]

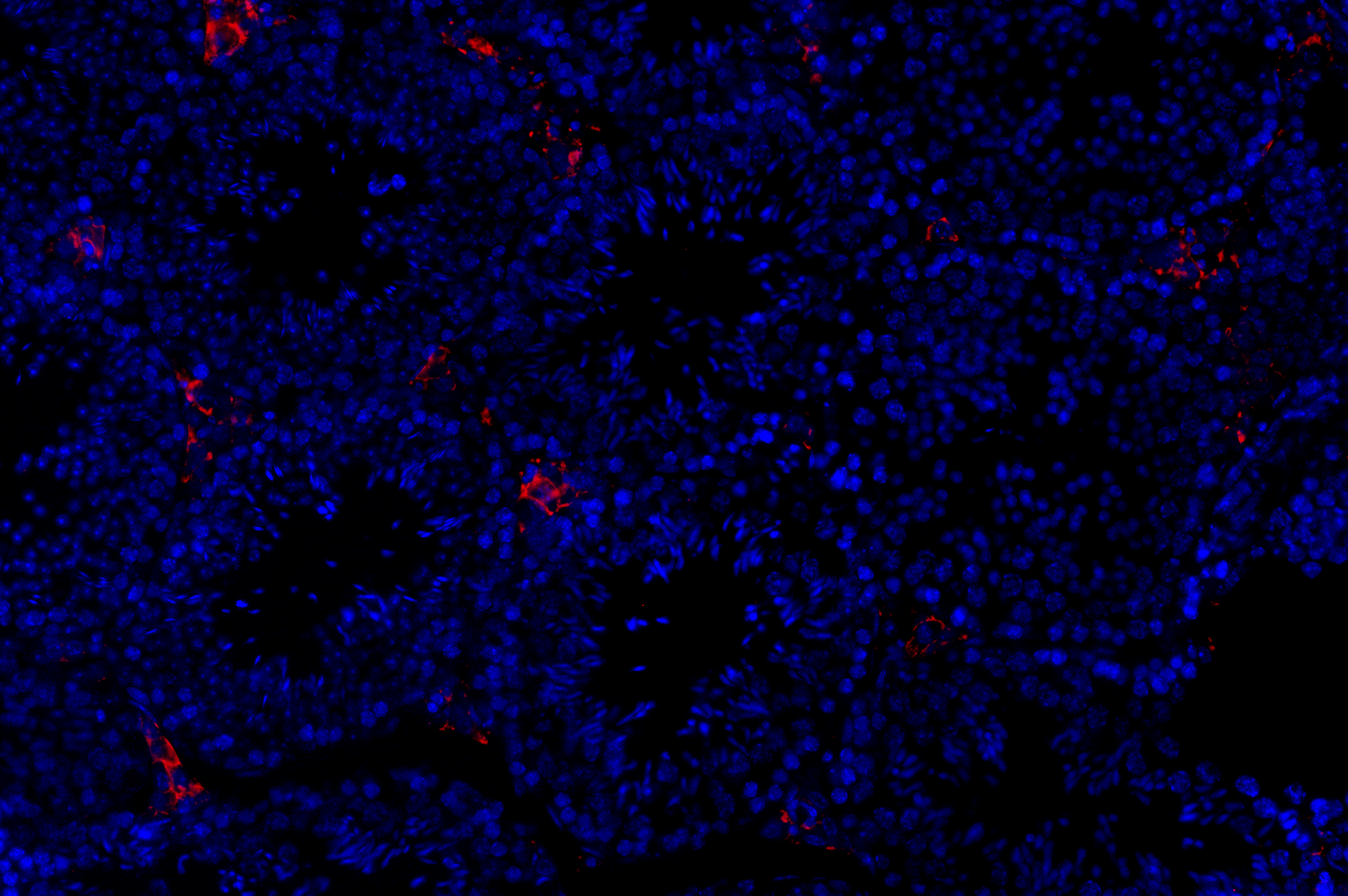

Supplement: Supplementary file 2 — Source Data Fig. 1 [file 44321_2023_16_MOESM2_ESM.zip › Figure1/Figure1D Micr image/caspase1 DAPI sham.tif]

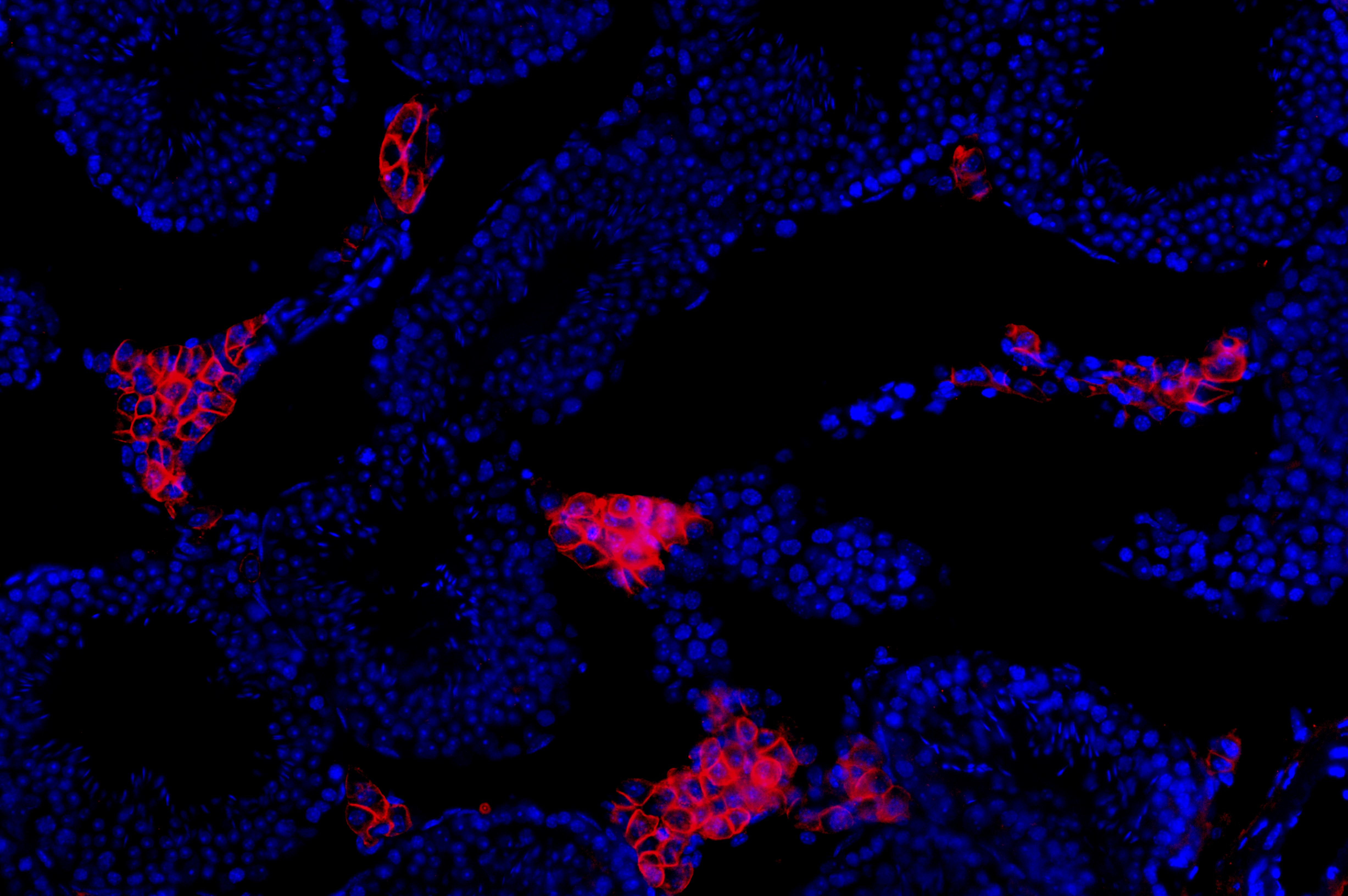

Supplement: Supplementary file 2 — Source Data Fig. 1 [file 44321_2023_16_MOESM2_ESM.zip › Figure1/Figure1D Micr image/caspase1 DAPI upec.tif]

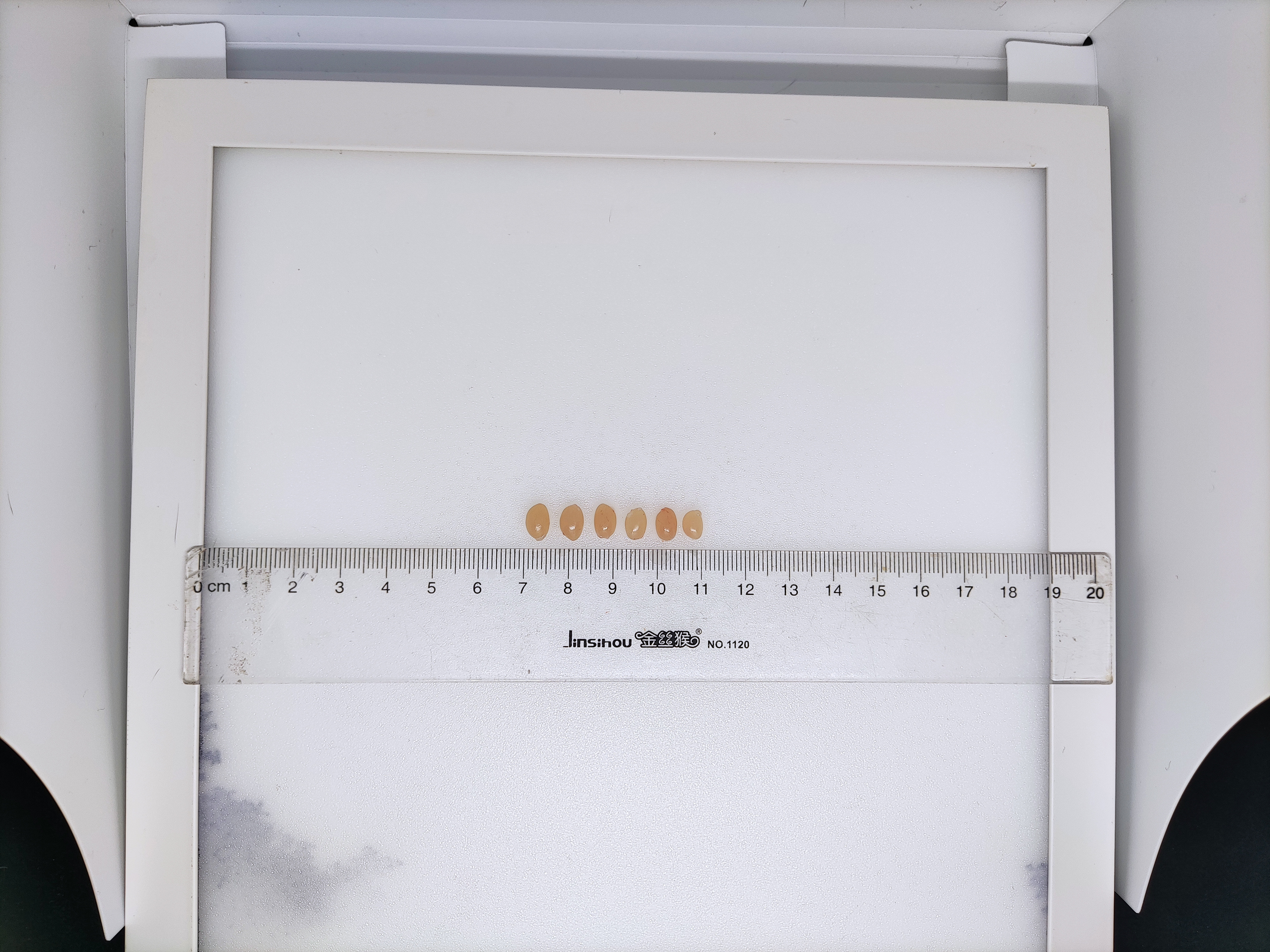

Supplement: Supplementary file 3 — Source Data Fig. 2 [file 44321_2023_16_MOESM3_ESM.zip › Figure2/Figure2A image and numerical data/image.tif]

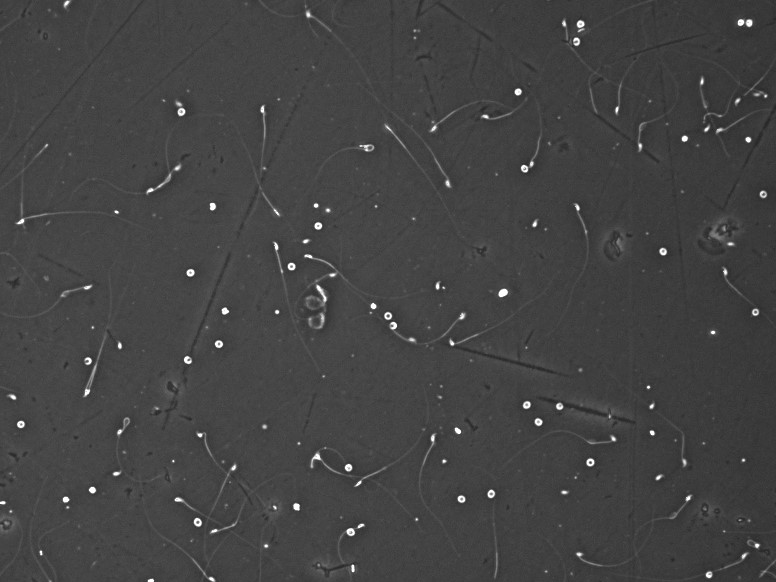

Supplement: Supplementary file 3 — Source Data Fig. 2 [file 44321_2023_16_MOESM3_ESM.zip › Figure2/Figure2C, D image and numerical data/ASCko.tif]

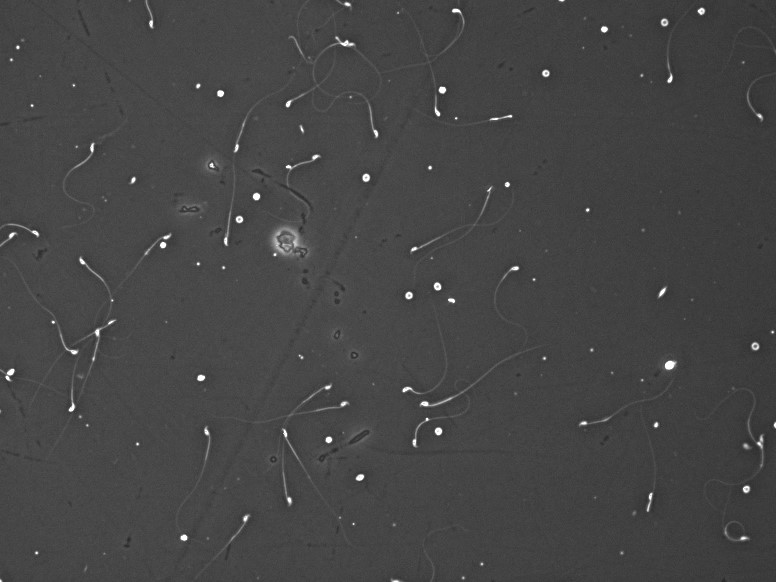

Supplement: Supplementary file 3 — Source Data Fig. 2 [file 44321_2023_16_MOESM3_ESM.zip › Figure2/Figure2C, D image and numerical data/aim2ko.tif]

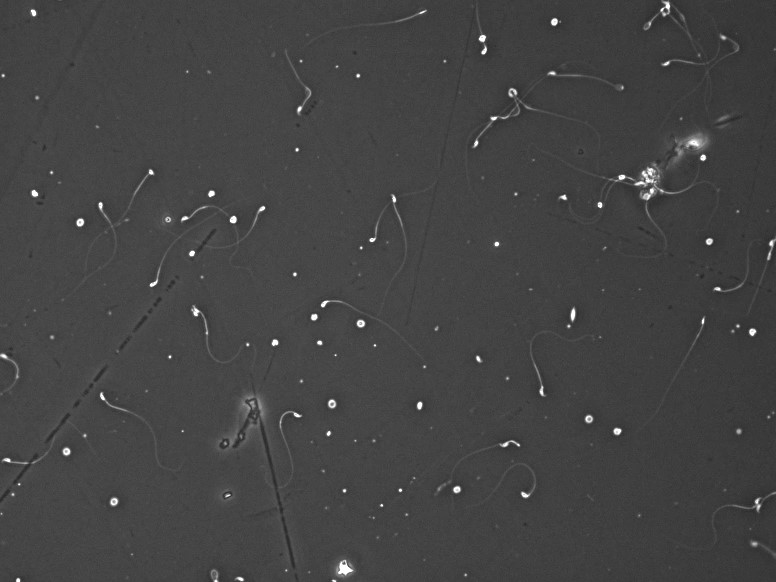

Supplement: Supplementary file 3 — Source Data Fig. 2 [file 44321_2023_16_MOESM3_ESM.zip › Figure2/Figure2C, D image and numerical data/casp1(11) ko.tif]

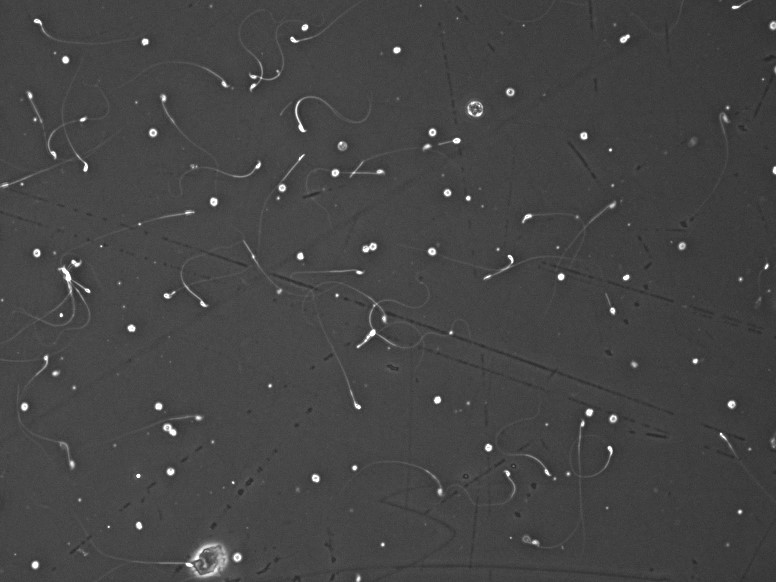

Supplement: Supplementary file 3 — Source Data Fig. 2 [file 44321_2023_16_MOESM3_ESM.zip › Figure2/Figure2C, D image and numerical data/gsdmd ko.tif]

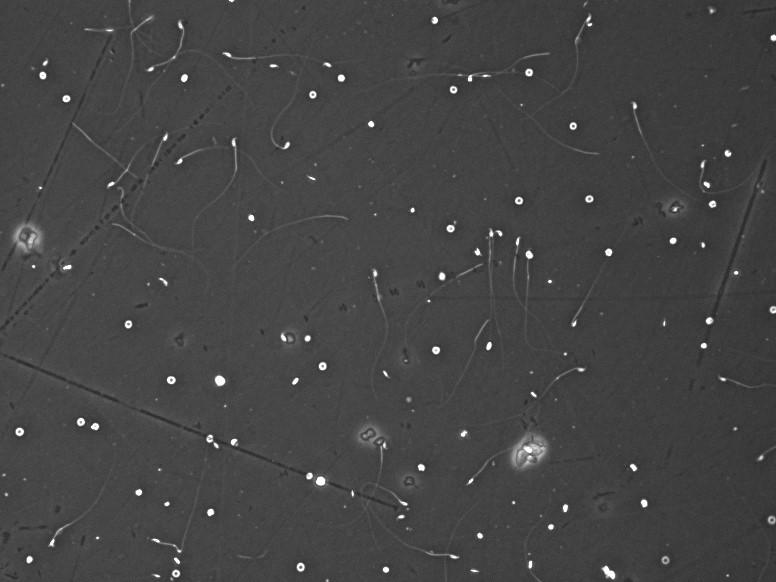

Supplement: Supplementary file 3 — Source Data Fig. 2 [file 44321_2023_16_MOESM3_ESM.zip › Figure2/Figure2C, D image and numerical data/nlrp3ko.tif]

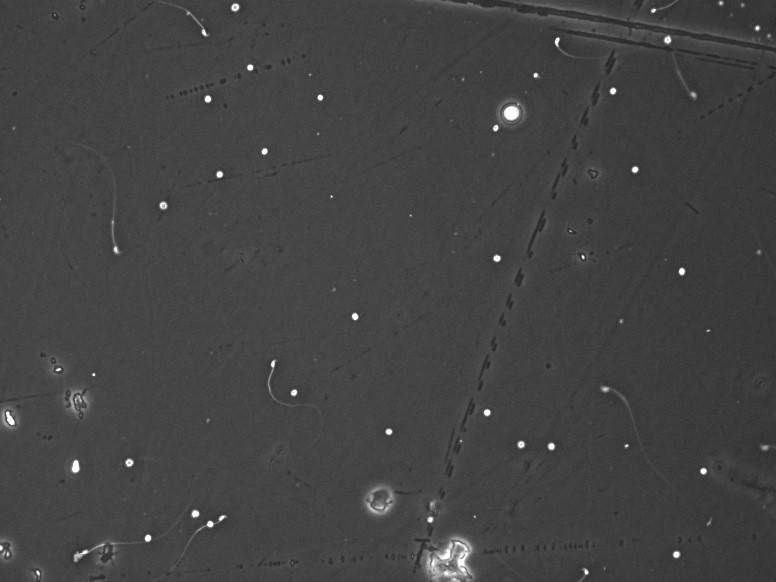

Supplement: Supplementary file 3 — Source Data Fig. 2 [file 44321_2023_16_MOESM3_ESM.zip › Figure2/Figure2C, D image and numerical data/wt.tif]

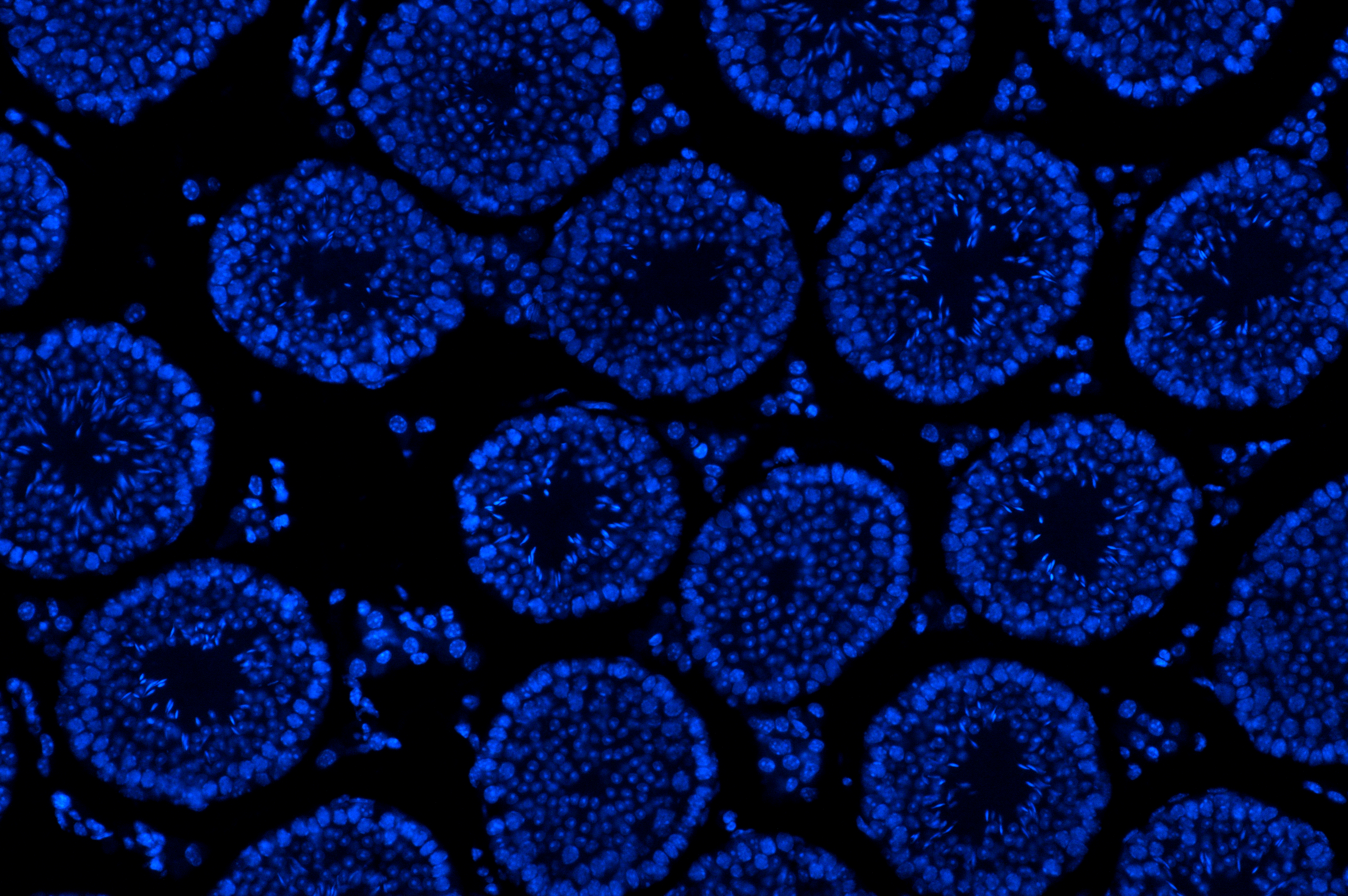

Supplement: Supplementary file 4 — Source Data Fig. 3 [file 44321_2023_16_MOESM4_ESM.zip › Figure3/Figure3B/DAPI Sham.tif]

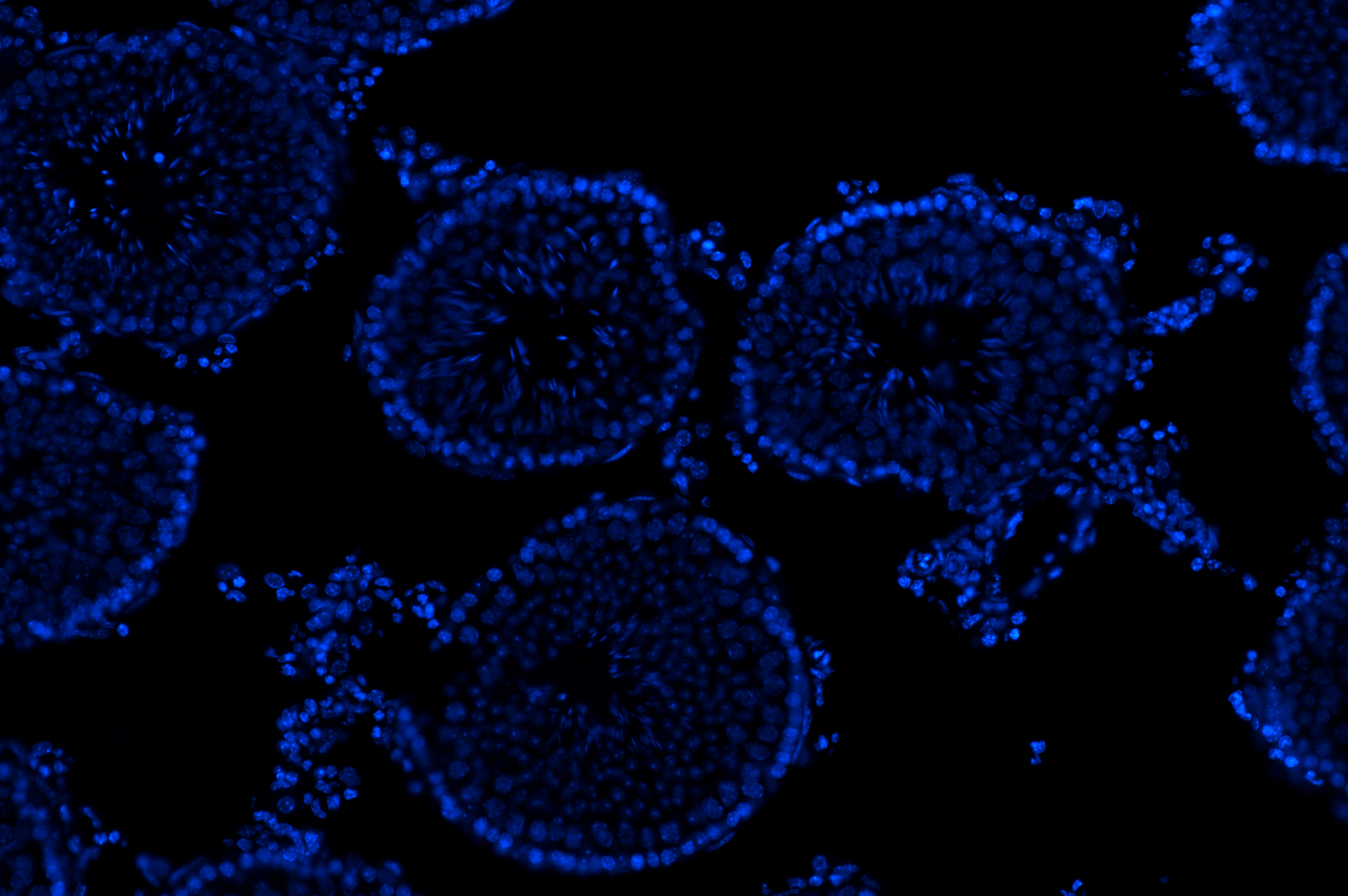

Supplement: Supplementary file 4 — Source Data Fig. 3 [file 44321_2023_16_MOESM4_ESM.zip › Figure3/Figure3B/DAPI UPEC.tif]

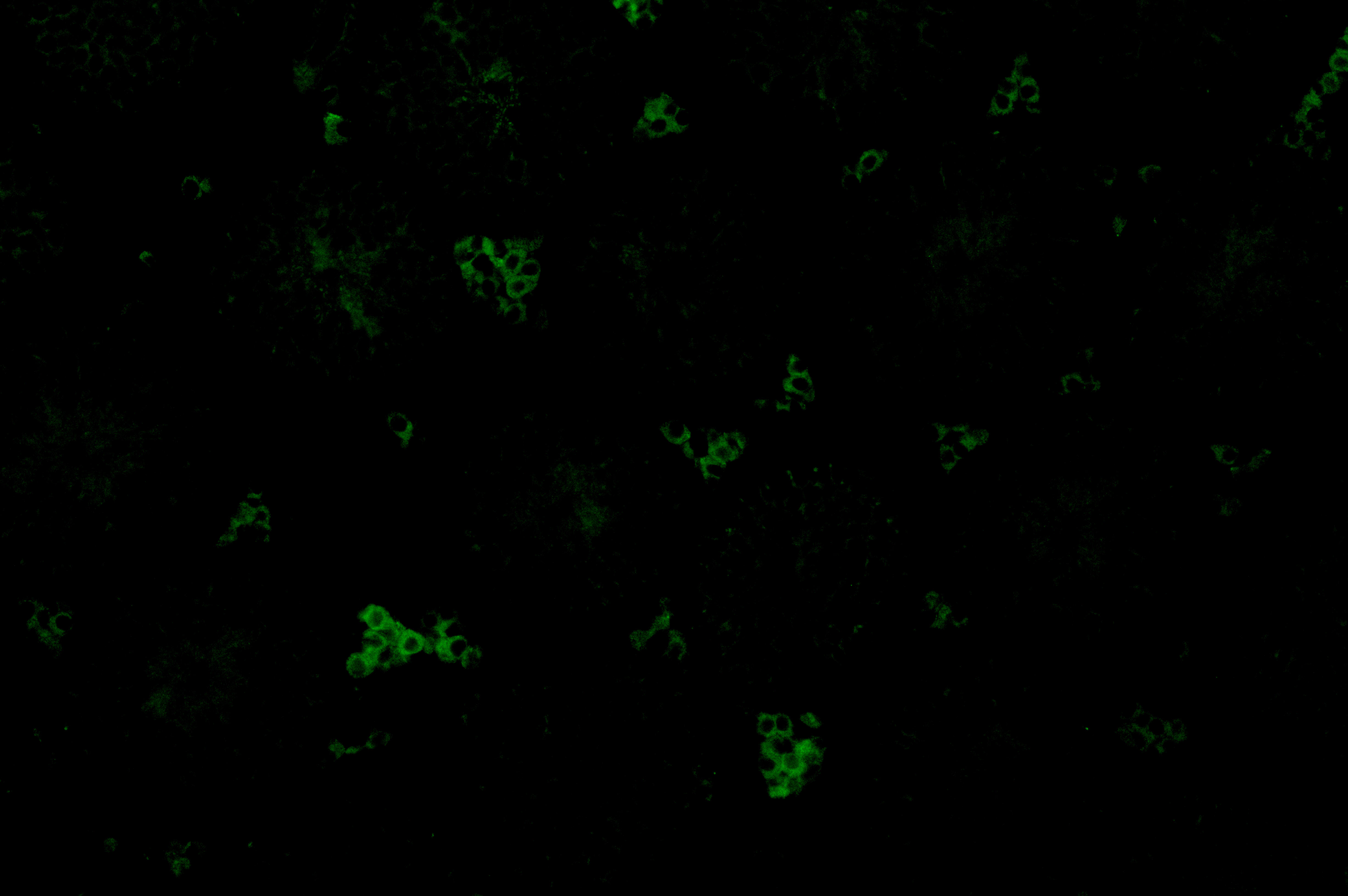

Supplement: Supplementary file 4 — Source Data Fig. 3 [file 44321_2023_16_MOESM4_ESM.zip › Figure3/Figure3B/F480 Sham.tif]

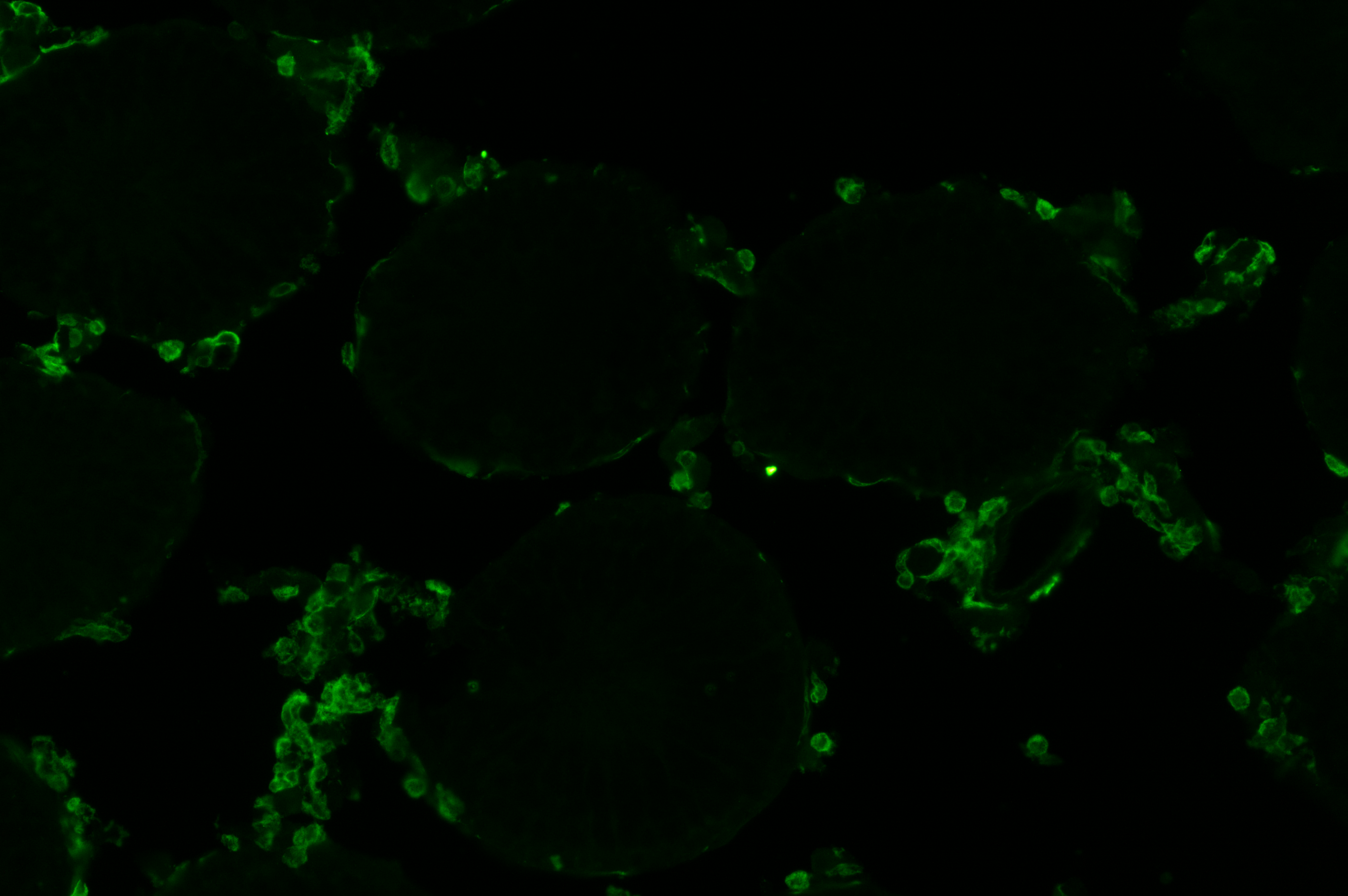

Supplement: Supplementary file 4 — Source Data Fig. 3 [file 44321_2023_16_MOESM4_ESM.zip › Figure3/Figure3B/F480 UPEC.tif]

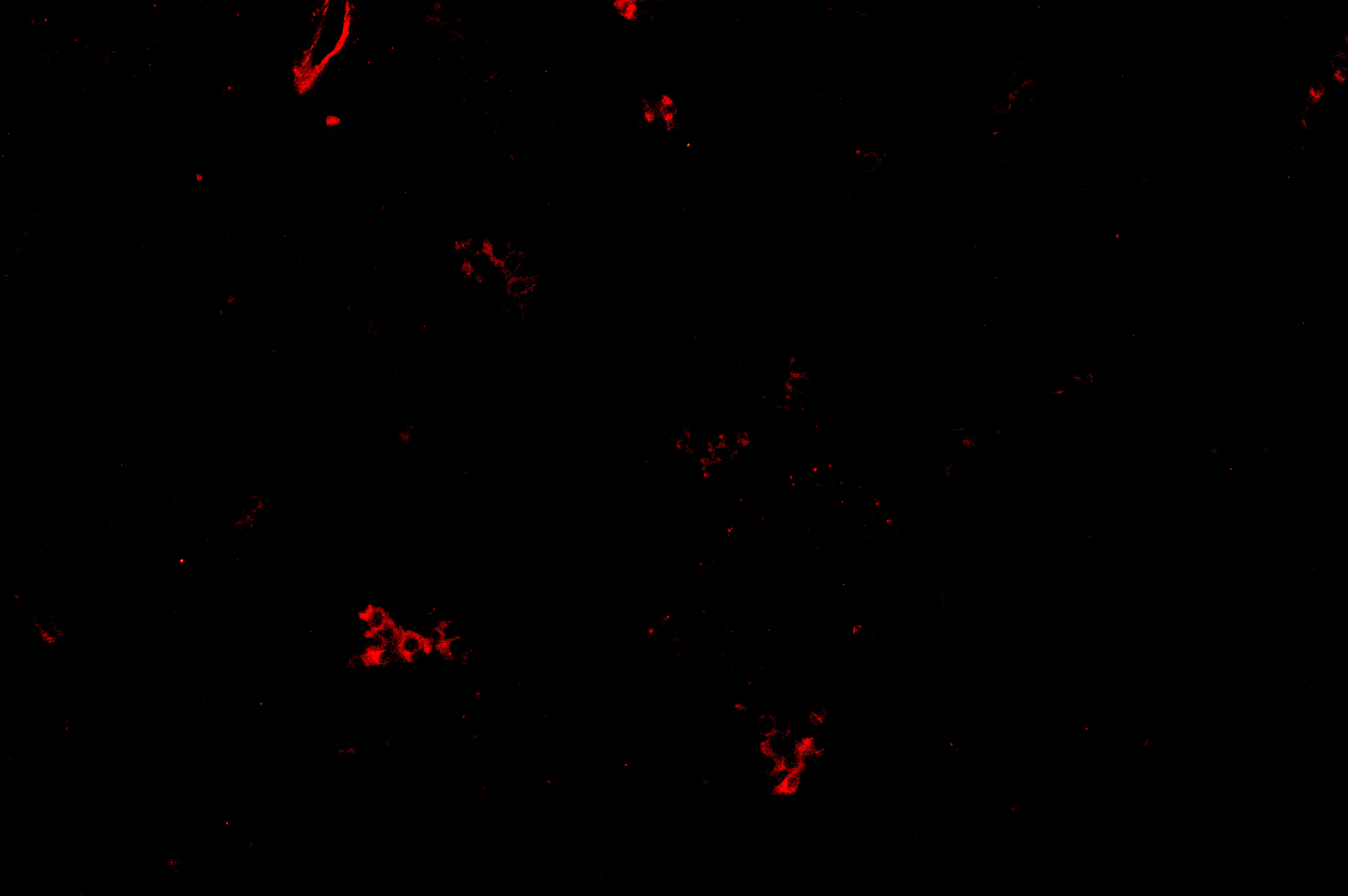

Supplement: Supplementary file 4 — Source Data Fig. 3 [file 44321_2023_16_MOESM4_ESM.zip › Figure3/Figure3B/GDSMD Sham.tif]

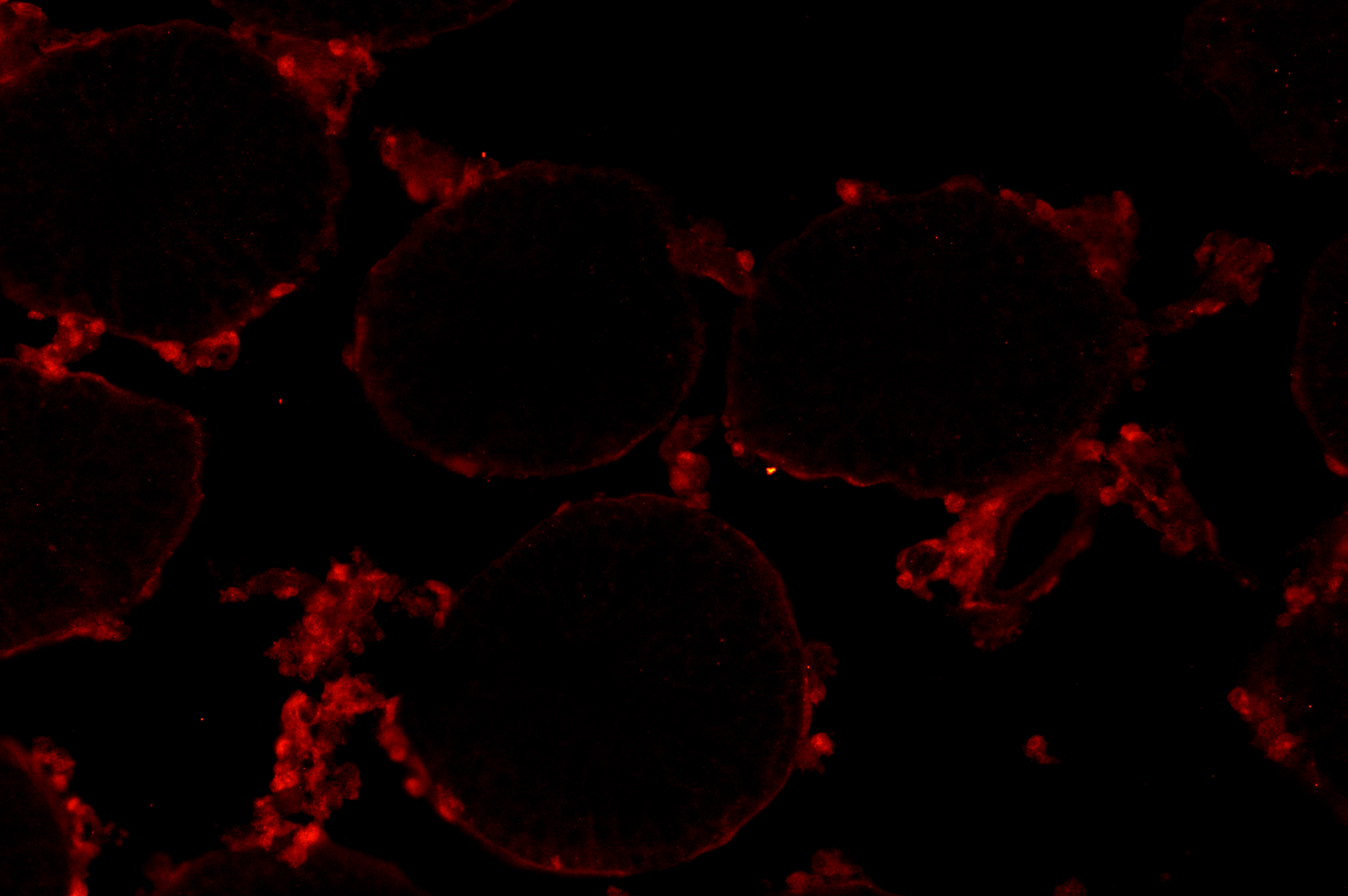

Supplement: Supplementary file 4 — Source Data Fig. 3 [file 44321_2023_16_MOESM4_ESM.zip › Figure3/Figure3B/GSDMD UPEC.tif]

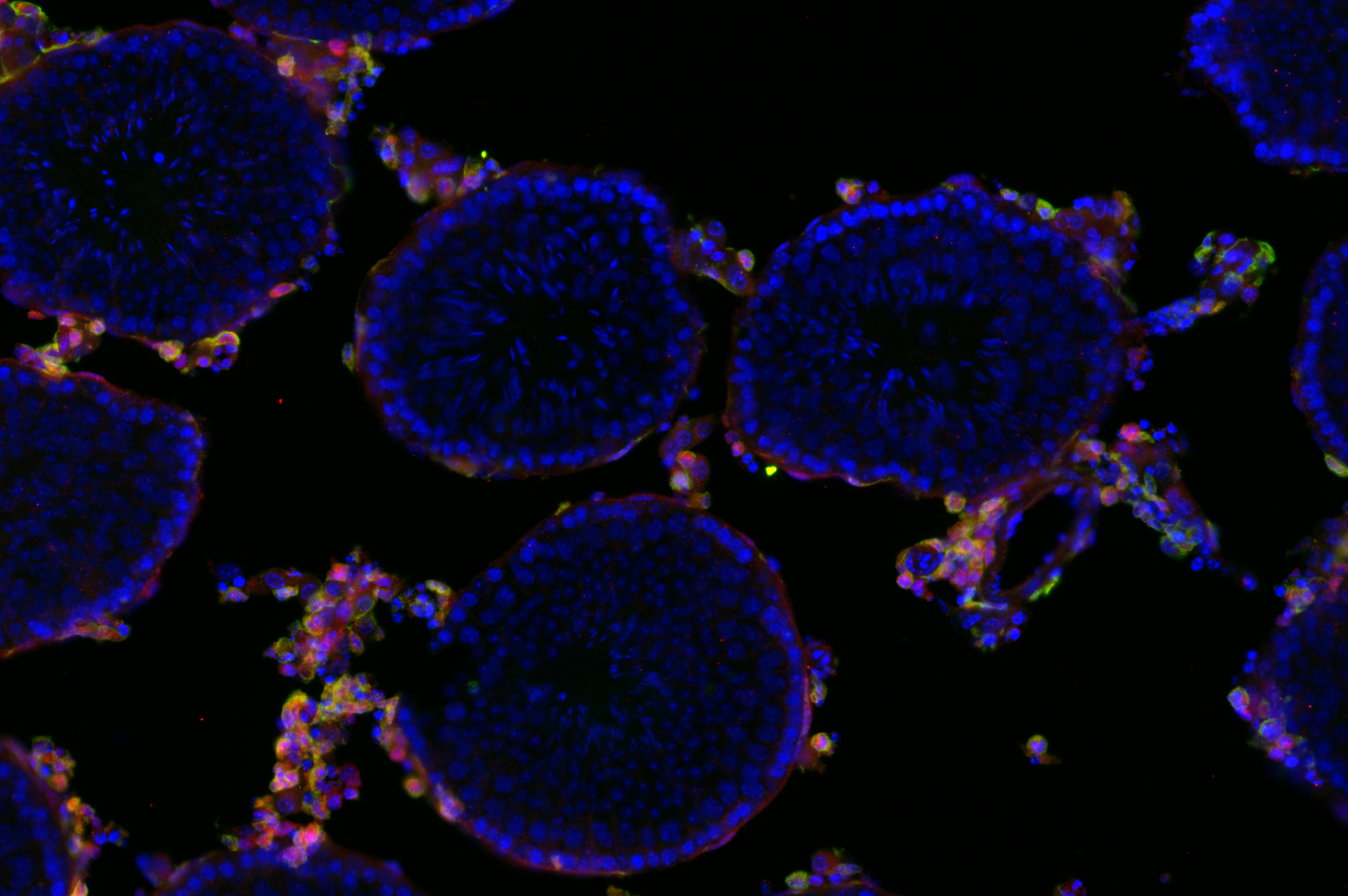

Supplement: Supplementary file 4 — Source Data Fig. 3 [file 44321_2023_16_MOESM4_ESM.zip › Figure3/Figure3B/Merge UPEC.tif]

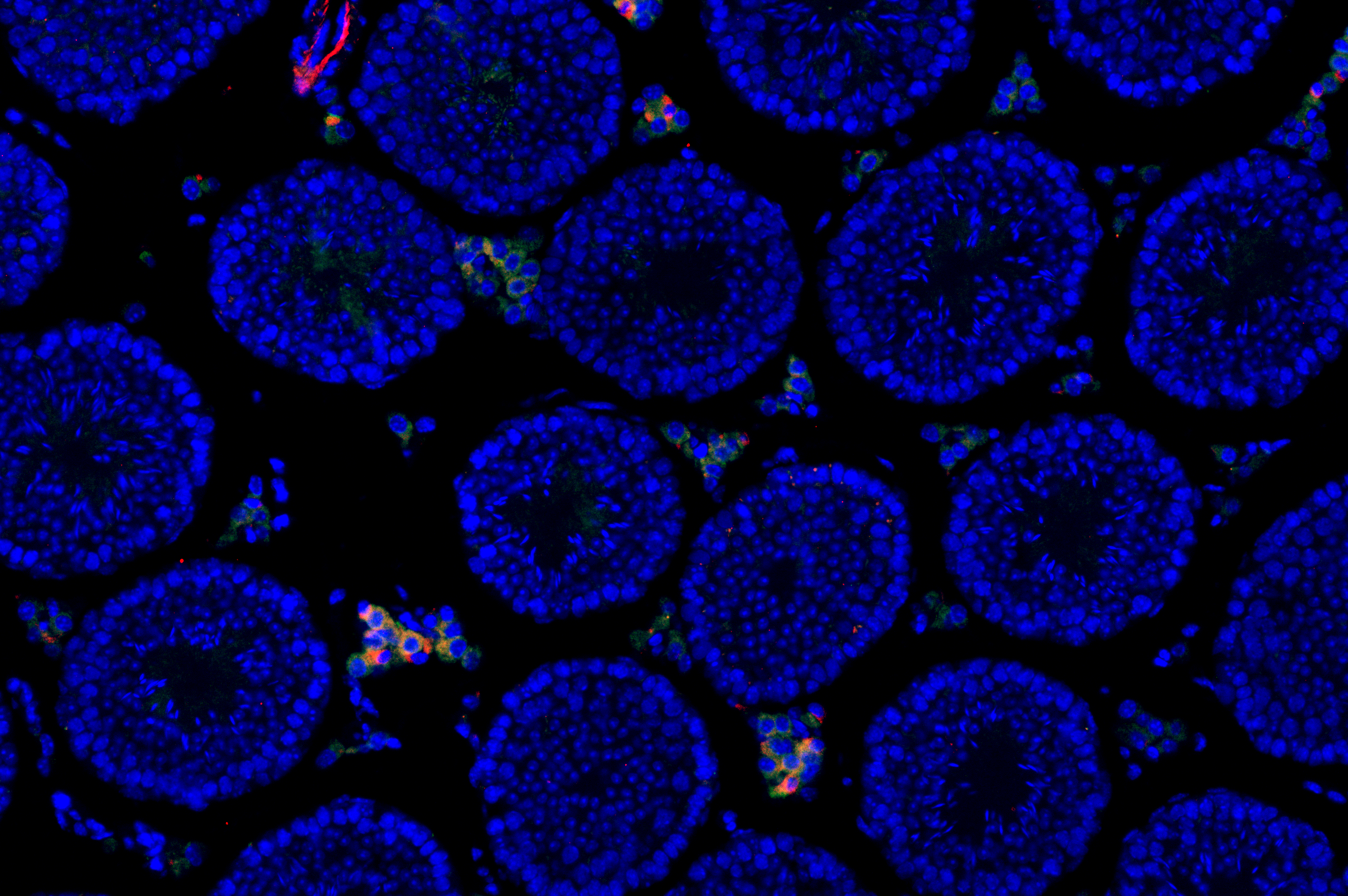

Supplement: Supplementary file 4 — Source Data Fig. 3 [file 44321_2023_16_MOESM4_ESM.zip › Figure3/Figure3B/merge.tif]

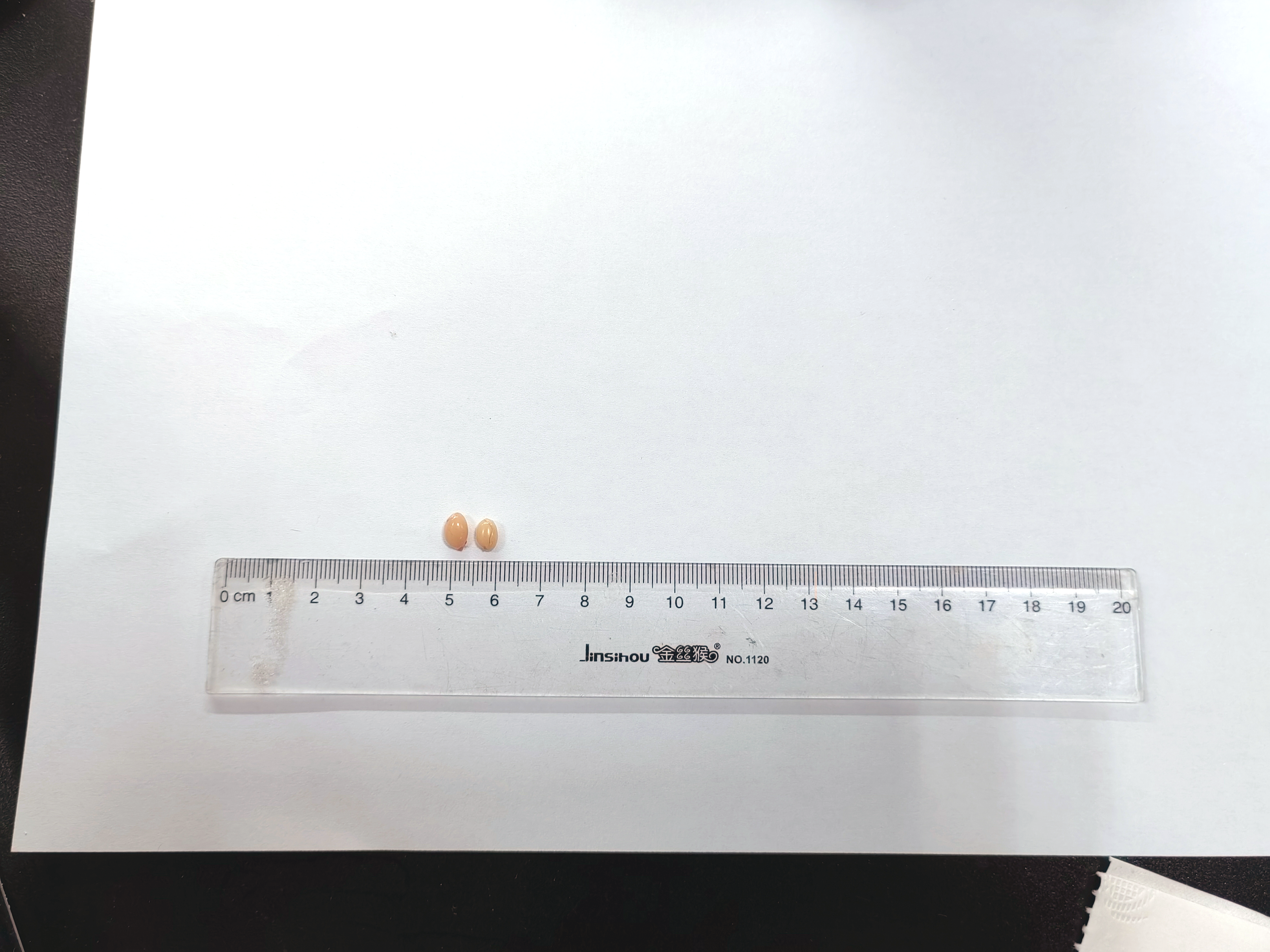

Supplement: Supplementary file 4 — Source Data Fig. 3 [file 44321_2023_16_MOESM4_ESM.zip › Figure3/Figure3C/testis.tif]

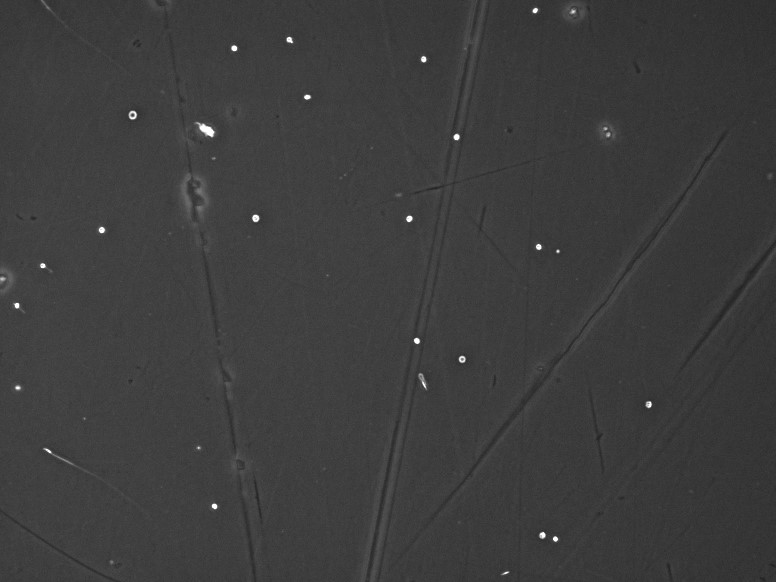

Supplement: Supplementary file 4 — Source Data Fig. 3 [file 44321_2023_16_MOESM4_ESM.zip › Figure3/Figure3E/Cx3cr1cre.tif]

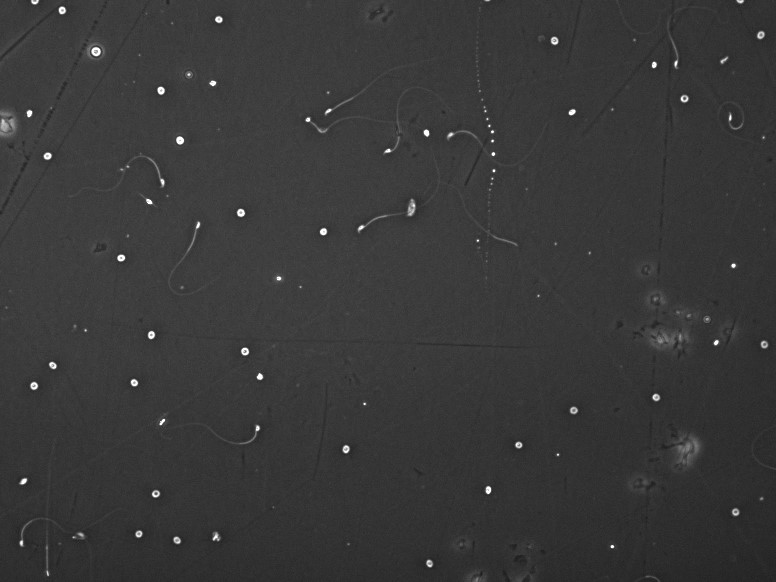

Supplement: Supplementary file 4 — Source Data Fig. 3 [file 44321_2023_16_MOESM4_ESM.zip › Figure3/Figure3E/Gsdmdfl Cx3cr1cre.tif]

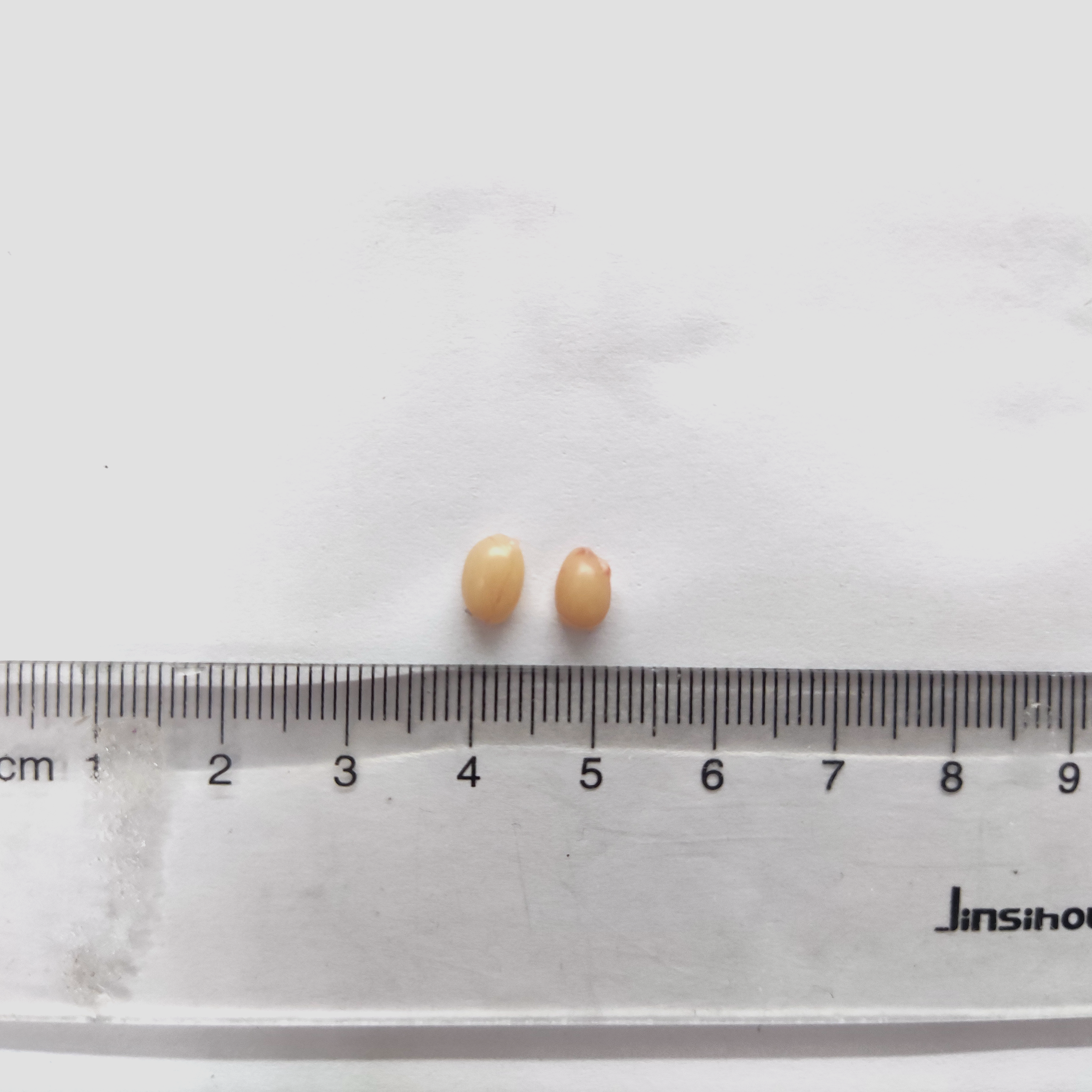

Supplement: Supplementary file 8 — Source Data Fig. 7 [file 44321_2023_16_MOESM8_ESM.zip › Figure7/Figure7B/testis.tif]

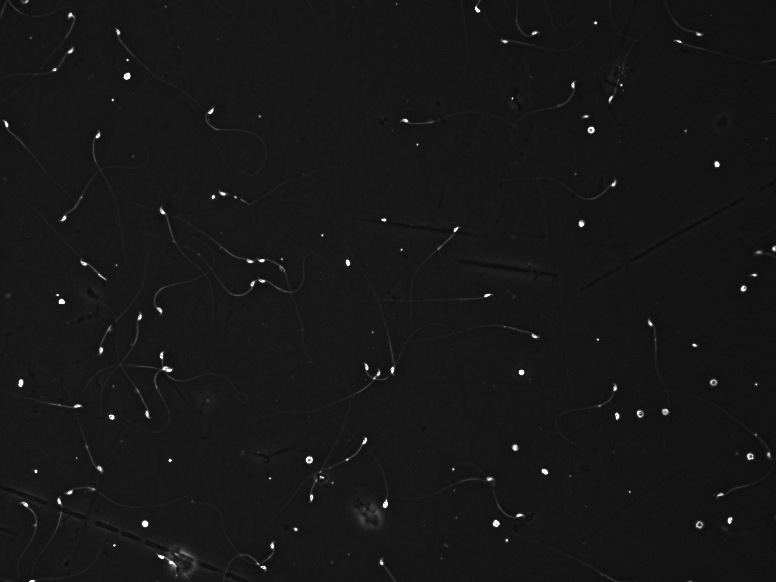

Supplement: Supplementary file 8 — Source Data Fig. 7 [file 44321_2023_16_MOESM8_ESM.zip › Figure7/Figure7D,E/mdf wt.tif]

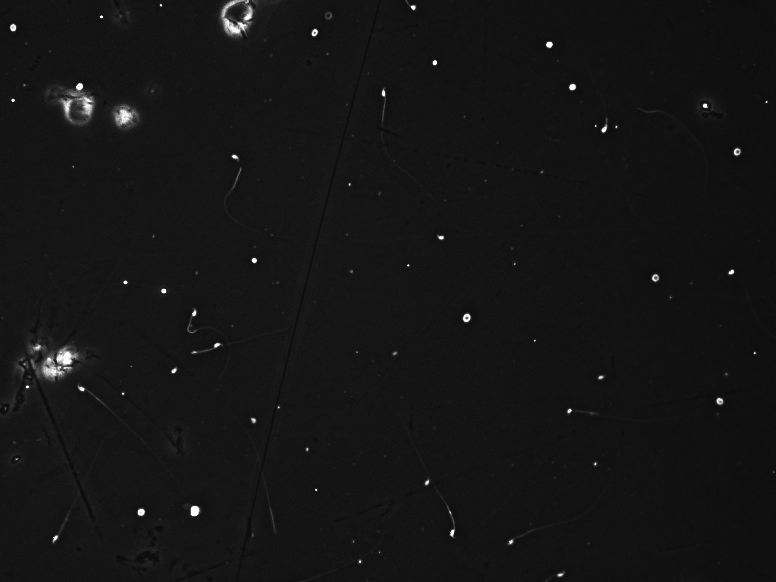

Supplement: Supplementary file 8 — Source Data Fig. 7 [file 44321_2023_16_MOESM8_ESM.zip › Figure7/Figure7D,E/wt.tif]

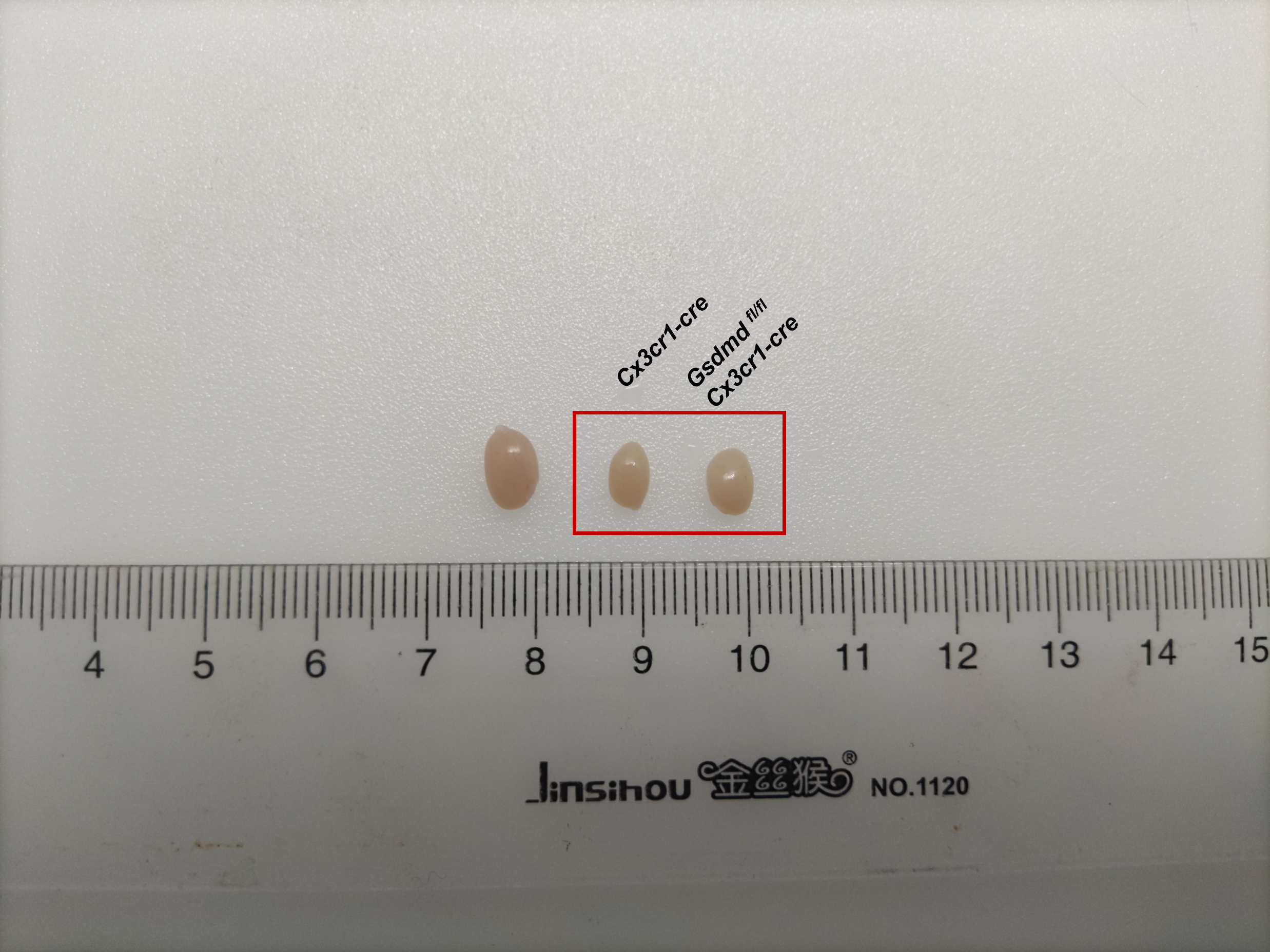

Supplement: Supplementary file 9 — Source Data Fig. 8 [file 44321_2023_16_MOESM9_ESM.zip › Figure8/Figure8B/testis.tif]

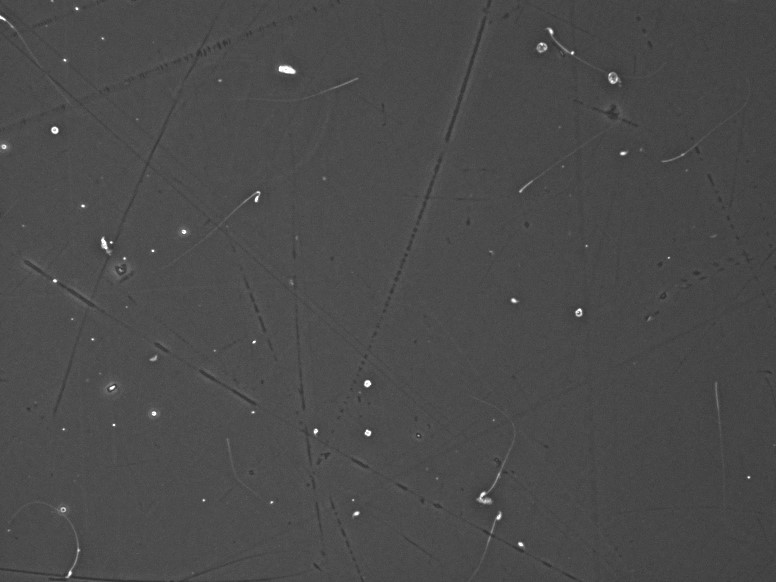

Supplement: Supplementary file 9 — Source Data Fig. 8 [file 44321_2023_16_MOESM9_ESM.zip › Figure8/Figure8D/EAO Cx3cr1cre.jpg]

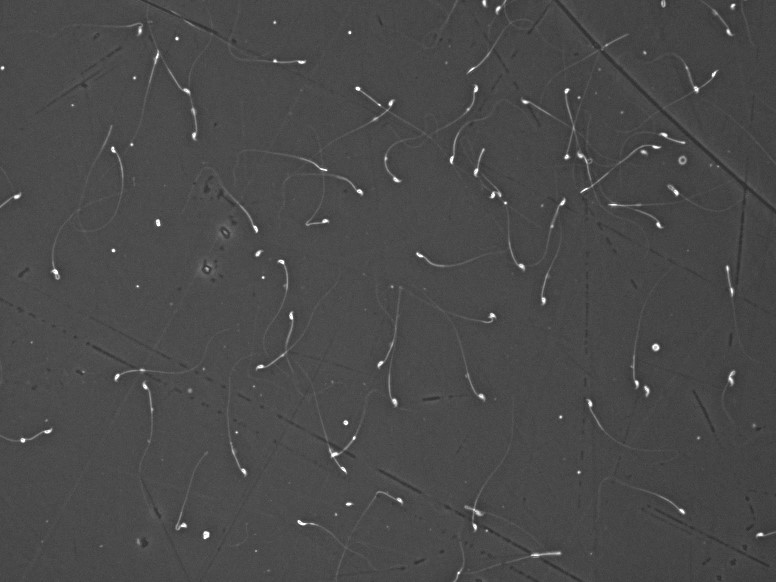

Supplement: Supplementary file 9 — Source Data Fig. 8 [file 44321_2023_16_MOESM9_ESM.zip › Figure8/Figure8D/EAO Gsdmdfl Cx3cr1cre.jpg]
