# Supplementary material for: Gasdermin D in macrophages drives orchitis by regulating inflammation and antigen presentation processes
Source: EMBO Mol Med. 2024 Jan 2;16(2):8. doi: 10.1038/s44321-023-00016-8 (PMC10897472; doi:10.1038/s44321-023-00016-8)
Supplement: Supplementary file 10 — Expanded View Figures [file 44321_2023_16_MOESM10_ESM.pdf]

Expanded View Figures

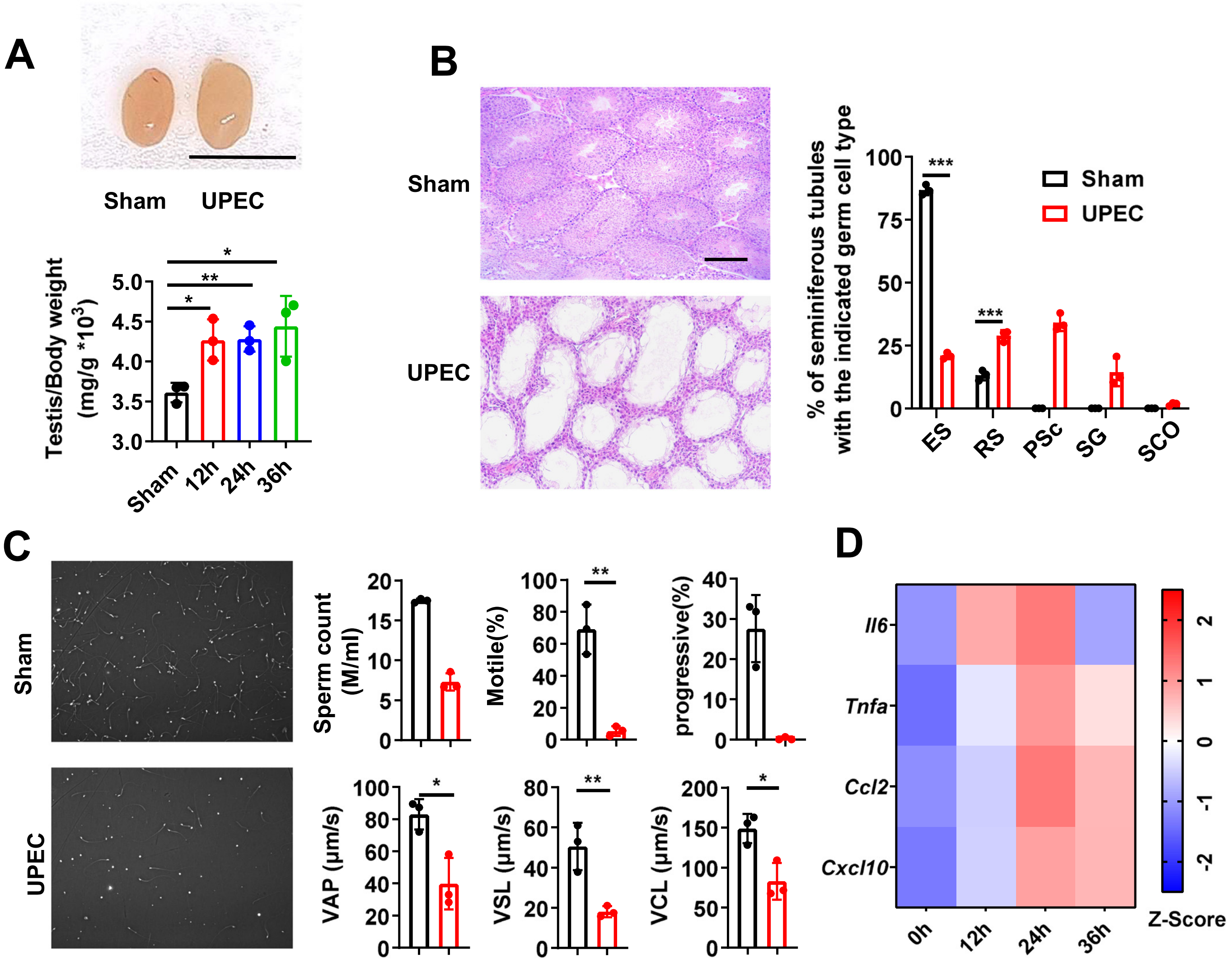

Figure EV1. UPEC can successfully induce acute orchitis.

(**A**) Gross morphology of representative testes from sham control mice and UPEC-induced orchitic mice, and relative weight of testis (testis weight (mg)/body weight (g) *10^3). Scale bars, 1 cm, ($n = 3$ mice per group). (**B**) Testicular histological changes were detected using H&E staining and the adaption of classical Johnsen scoring system, elongated spermatids (ES), round spermatids (RS), pachytene spermatocytes (PSc), spermatogonia (SG), Sertoli-cell-only (SCO). Scale bars, 100 μm, ($n = 3$ mice per group). (**C**) Sperm count, ratio of motile and progressive sperm, and velocity of motile sperm (VAP, average path velocity; VSL, straight-line velocity; VCL, curvilinear velocity) were detected using computer-assisted sperm analysis (CASA), ($n = 3$ mice per group). (**D**) Relative mRNA levels of *Il-6*, *Tnfa*, *Ccl-2*, and *Cxcl-10* in the testes of mice treated with UPEC (1×10^5 CFU) for indicated time ($n = 3$ mice per group). Data information: Data are pooled from two independent experiments. Error bars show mean ± s.d. *$P < 0.05$, **$P < 0.01$. Two-tailed unpaired Student's *t* test was used unless otherwise stated.

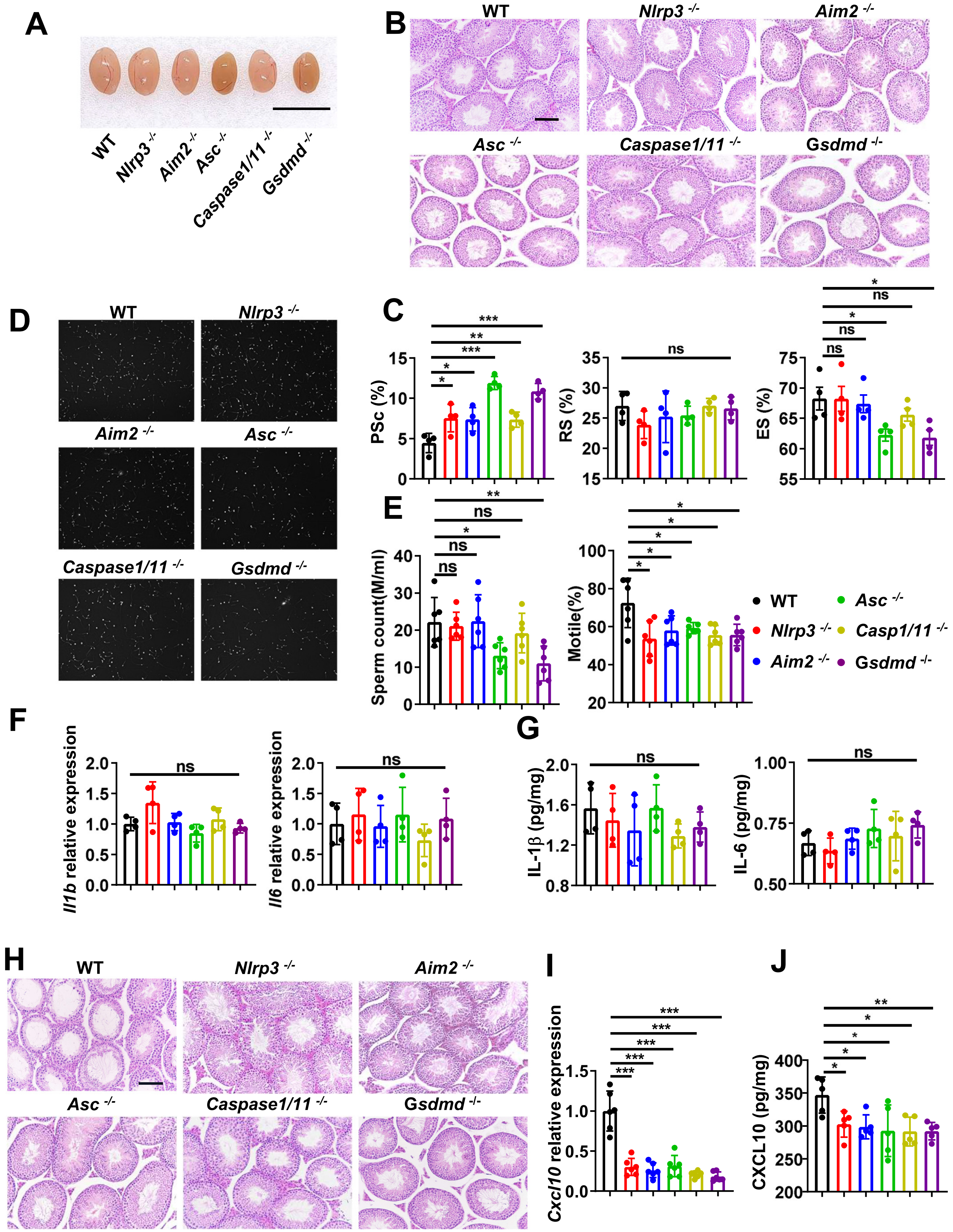
A
WT
Nlrp3 -/-
Aim2 -/-
Asc -/-
Caspase1/11 -/-
Gsdmd -/-
B
WT
Nlrp3 -/-
Aim2 -/-
Asc -/-
Caspase1/11 -/-
Gsdmd -/-
C
PSc (%)
RS (%)
ES (%)
ns
*
**
***
D
WT
Nlrp3 -/-
Aim2 -/-
Asc -/-
Caspase1/11 -/-
Gsdmd -/-
E
Sperm count(M/ml)
Motile(%)
WT
Nlrp3 -/-
Aim2 -/-
Asc -/-
Casp1/11 -/-
Gsdmd -/-
F
Il1b relative expression
Il6 relative expression
G
IL-1β (pg/mg)
IL-6 (pg/mg)
H
WT
Nlrp3 -/-
Aim2 -/-
Asc -/-
Caspase1/11 -/-
Gsdmd -/-
I
Cxcl10 relative expression
J
CXCL10 (pg/mg)

 © The Author(s)

Figure EV2. Deletion of GSDMD did not induce spontaneous orchitis.

Testicular tissues from age-matched male WT, *Nlrp3*$^{-/-}$, *Aim2*$^{-/-}$, *Asc*$^{-/-}$, *Caspase-1/11*$^{-/-}$, *and Gsdmd*$^{-/-}$ mice were collected for subsequent experiments. (**A**) Testicular morphology. Scale bars, 1 cm. (**B**) Representative images of H&E staining of testes from the mice in (**A**). (**C**) Testicular histological changes of testes in (**B**) were calculated using the adaption of classical Johnsen scoring system. Scale bars, 100 μm, ($n = 4$ mice per group). (**D**) Representative CASA picture of sperm status of indicated mice. (**E**) Sperm count, ratio of motile sperm of indicated mice were detected using CASA ($n = 6$ mice per group). (**F**) Relative mRNA levels of *Il1b* and *Il-6* in the testes of indicated mice ($n = 4$ mice per group). (**G**) ELISA analysis of IL-1β and IL-6 levels in the testes of the indicated mice ($n = 4$ mice per group). (**H**) Representative images of H&E staining of testes from the indicated mice treated with UPEC for 7 days. (**I**) Relative mRNA levels of *Cxcl-10* in the testes from the indicated mice treated with UPEC for 24 h ($n = 6$ mice per group). (**J**) ELISA analysis of CXCL-10 in the testes from the indicated mice treated with UPEC for 36 h ($n = 6$ mice per group). Data information: Data are pooled from three independent experiments for (**C**, **E–G**, **I**, **J**). Data are representative of three independent experiments for (**A**, **B**, **D**), and (**H**) error bars show mean ± s.d. *$P < 0.05$, **$P < 0.01$, ***$P < 0.001$, ns, not significant. Two-tailed unpaired Student's *t* test was used unless otherwise stated. Mann–Whitney test for comparisons of relative expression of *Il-6* between WT and *Caspase-1/11*$^{-/-}$ in (**F**), and Mann–Whitney test for comparisons of CXCL-10 level between WT and *Caspase-1/11*$^{-/-}$ in (**J**).

© The Author(s)

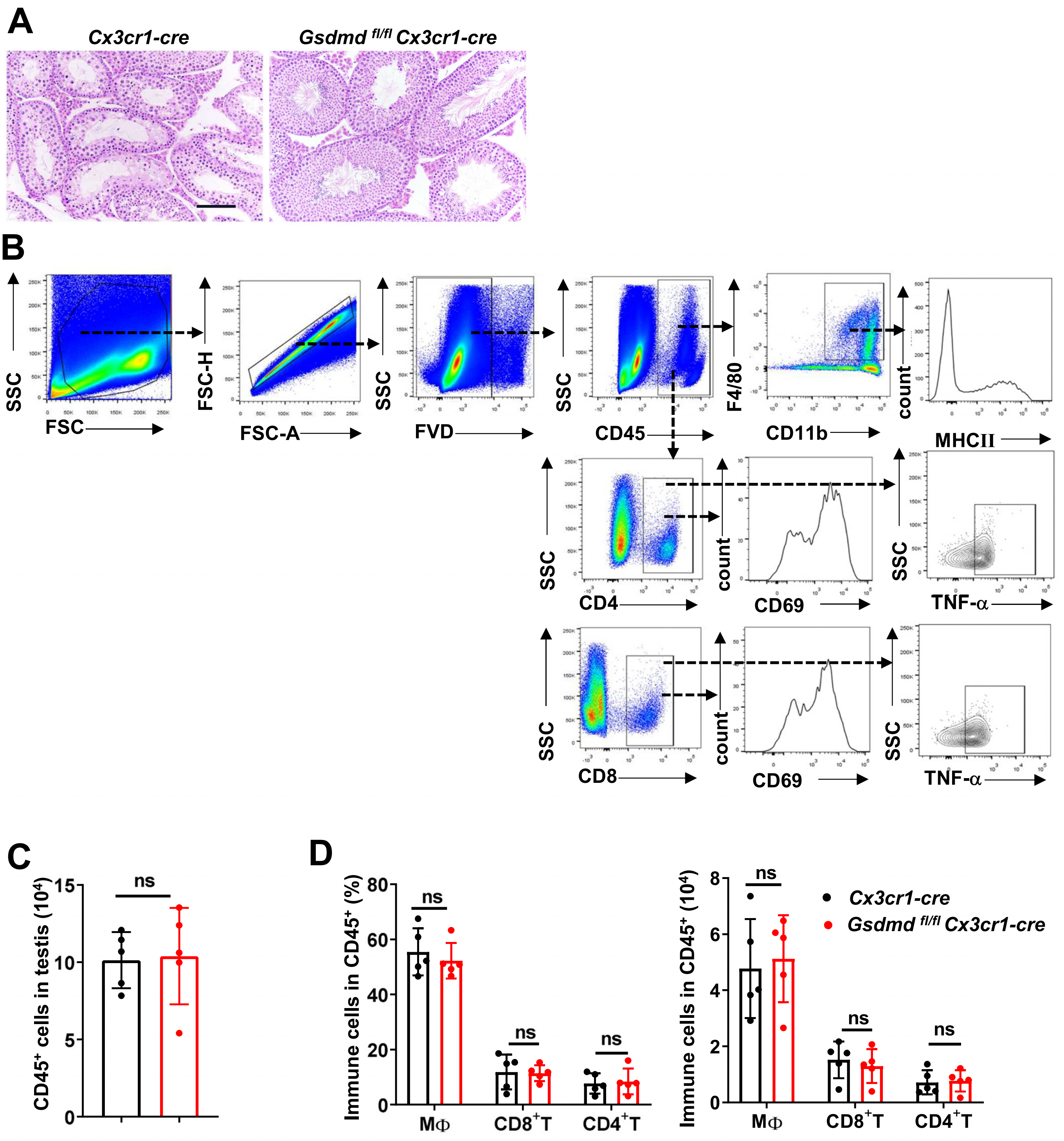

Figure EV3. GSDMD deficiency in macrophages alleviated testicular inflammation during UPEC-induced acute orchitis.

(**A**) Representative images of H&E staining of testes from *Gsdmd*$^{fl/fl}$*Cx3cr1*-cre and *Cx3cr1*-cre mice treated with UPEC for 7 days. (**B**) Flow cytometric gating strategy for Fig. 4. (**C, D**) Testes from *Gsdmd*$^{fl/fl}$*Cx3cr1*-cre and *Cx3cr1*-cre mice aged at 10–12 weeks were collected for flow cytometric analysis. The number of total immune cells (**C**) and the quantified absolute number (**D**) of the macrophages ($CD11b^+F4/80^+$), $CD8^+$ T cells, $CD4^+$ T cells, ($n = 5$ mice per group). Data information: Error bars show mean ± s.d. ns, not significant. Two-tailed unpaired Student's *t* test was used unless otherwise stated.

 © The Author(s)

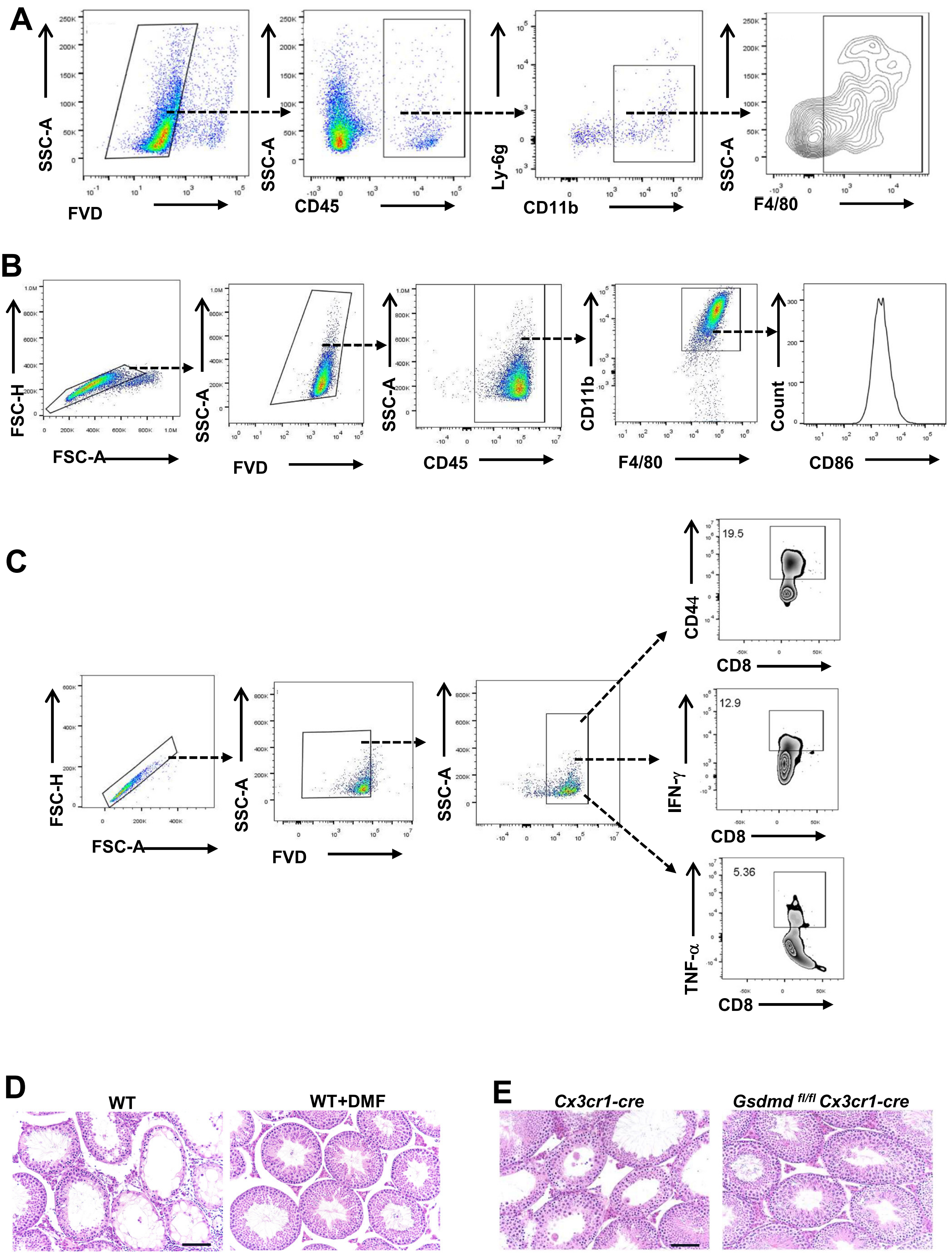
A
SSC-A
FVD
CD45
Ly-6g
CD11b
F4/80
B
FSC-H
FSC-A
SSC-A
FVD
CD45
CD11b
F4/80
Count
CD86
C
FSC-H
FSC-A
SSC-A
FVD
CD44
CD8
19.5
IFN-γ
12.9
TNF-α
5.36
D
WT
WT+DMF
E
Cx3cr1-cre
Gsdmd fl/fl Cx3cr1-cre

Figure EV4. FACS gating strategy.

(**A**) FACS gating strategy for sorting testicular macrophage in Fig. 5. (**B**, **C**) FCM gating strategy for analysis of BMDMs (**B**) and T cells (**C**) in Fig. 6. (**D**) Testicular histological changes of testes in UPEC-induced orchitic WT mice injected vehicle or DMF were detected by H&E staining. Scale bars, 100 μm. (**E**) Testicular histological changes of testes in *Gsdmd*$^{fl/fl}$*Cx3cr1*-cre and *Cx3cr1*-cre EAO mice were detected using H&E staining. Scale bars, 100 μm.

 © The Author(s)

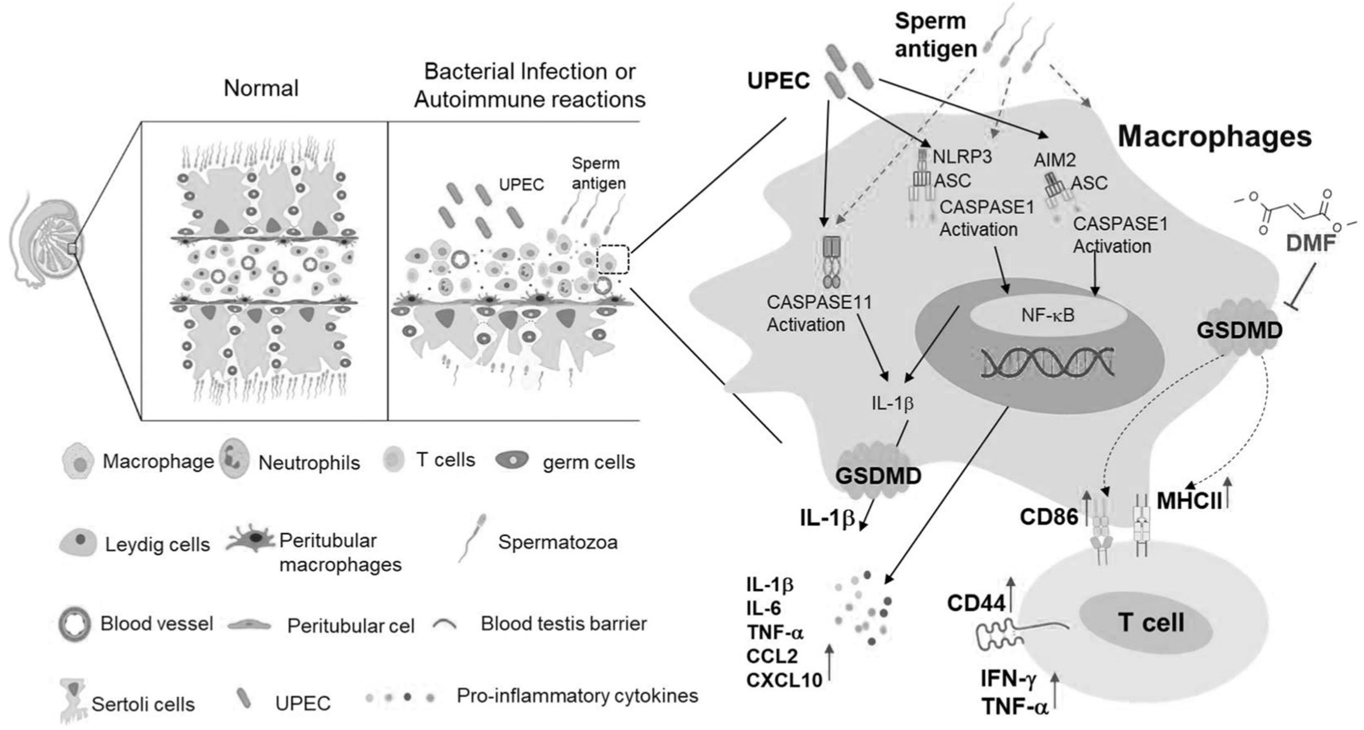

Figure EV5. Model showing how GSDMD in macrophages drives orchitis.

During orchitis, GSDMD and other inflammasome-related molecules are activated by UPEC or sperm antigens, thereby promoting antigen presentation and inflammatory responses in testicular macrophages to enhance T-cell responses, which driven the development of acute and chronic orchitis. Administration of GSDMD inhibitors conferred protection against orchitis in mice.

© The Author(s)
